# Prevalence and patient characteristics associated with cardiovascular disease risk factor screening in UK primary care for people with severe mental illness: an electronic healthcare record study

**DOI:** 10.1136/bmjment-2024-301409

**Published:** 2025-01-15

**Authors:** Naomi Launders, Caroline Anne Jackson, Joseph F Hayes, Ann John, Robert Stewart, Matthew H Iveson, Elvira Bramon, Bruce Guthrie, Stewart William Mercer, David P J Osborn

**Affiliations:** 1 Division of Psychiatry, University College London, London, UK; 2 The University of Edinburgh Usher Institute, Edinburgh, UK; 3 Camden and Islington NHS Foundation Trust, London, UK; 4 Swansea University, Swansea, UK; 5 Institute of Psychiatry, Psychology and Neuroscience, King's College London, London, UK; 6 South London and Maudsley NHS Foundation Trust, London, UK; 7 Division of Psychiatry, The University of Edinburgh Centre for Clinical Brain Sciences, Edinburgh, UK

**Keywords:** Schizophrenia & psychotic disorders

## Abstract

**Background:**

People with severe mental illness (SMI) are at increased risk of cardiovascular disease (CVD), and initiatives for CVD risk factor screening in the UK have not reduced disparities.

**Objectives:**

To describe the annual screening prevalence for CVD risk factors in people with SMI from April 2000 to March 2018, and to identify factors associated with receiving no screening and regular screening.

**Methods:**

We identified adults with a diagnosis of SMI (schizophrenia, bipolar disorder or ‘other psychosis’) from UK primary care records in Clinical Practice Research Datalink. We calculated the annual prevalence of screening for blood pressure, cholesterol, glucose, body mass index, alcohol consumption and smoking status using multinomial logistic regression to identify factors associated with receiving no screening and complete screening.

**Results:**

Of 216 136 patients with SMI, 55% received screening for all six CVD risk factors at least once during follow-up and 35% received all six within a 1-month period. Our findings suggest that patient characteristics and financial incentivisation influence screening prevalence of individual CVD risk factors, the likelihood of receiving screening for all six CVD risk factors annually and risk of receiving no screening.

**Conclusions:**

The low proportion of people with SMI receiving regular comprehensive CVD risk factor screening is concerning. Screening needs to be embedded as part of broad physical health checks to ensure the health needs of people with SMI are being met. If we are to improve cardiovascular health, interventions are needed where risk of receiving no screening or not receiving regular screening is highest.

**WHAT IS ALREADY KNOWN ON THIS TOPIC**

- The prevalence of cardiovascular disease (CVD) risk factor screening in primary care increased in people with severe mental illness (SMI) with the introduction of an incentivisation scheme, and there is some evidence that when incentivisation for screening for specific CVD risk factors was removed the screening decreased.

**WHAT THIS STUDY ADDS**

- Few patients received regular screening for all six CVD risk factors considered, and both screening prevalence and risk of receiving no screening varied depending on patient characteristics.
- Only 35% of patients ever received screening for all six CVD risk factors considered in a 1-month period, suggesting that screening is not often being done as part of a physical health check.

**HOW THIS STUDY MIGHT AFFECT RESEARCH, PRACTICE OR POLICY**

- Clinicians should be aware that some subgroups of patients with SMI are less likely to receive screening, and the importance of providing screening as part of a regular, comprehensive physical health check.

## Background

 People with severe mental illnesses (SMI), such as schizophrenia, bipolar disorder or other psychotic illnesses, are at increased risk of many physical health conditions.^1 2^ People with SMI have 1.5–2.5 times the risk of cardiovascular disease (CVD) compared with the general population and an increased risk of death from CVD.^3^,^6^ They also have a higher prevalence of CVD risk factors, such as smoking, obesity and diabetes,^17^,^9^ which is compounded by cardiometabolic side effects of antipsychotic medication,^10^ and sociodemographic risk factors.^11 12^

In recognition of physical health disparities in people with SMI, financial incentivisation of physical health checks for people with SMI in primary care was introduced in the UK in 2004 through the Quality and Outcomes Framework (QOF).^13^ QOF initially incentivised a review of physical health, with incentivisation of screening for individual CVD risk factors introduced in 2011. While blood pressure and alcohol consumption screening has been consistently incentivised since 2011 and smoking status has been incentivised since 2008, cholesterol, glucose and body mass index (BMI) screening has been less consistently incentivised (see online supplemental table S1 for QOF changes related to SMI). Additionally, from 2014 the measures incentivised differed across the constituent countries of the UK, and Scotland and Wales abolished QOF in 2016 and 2019, respectively.

Several studies have shown an increase in recording of CVD risk factors following the introduction of QOF in people with SMI,^14^,^17^ and a recent cohort study found that in England, removal of cholesterol and BMI as incentivised indicators resulted in a decrease in recording of these risk factors compared with blood pressure recording.^18^ However, there is a lack of evidence regarding long-term trends in screening prevalence for the six CVD risk factors currently included in the National Health Service (NHS) England Physical Health Check for SMI and patient characteristics associated with receiving screening. In order to identify unmet needs and improve the health of people with SMI, it is important to understand whether incentivisation drives increases in screening in all patients with SMI, and to identify which individuals may be at risk of not being screened.

Objective: To investigate the long-term trends and patient characteristics associated with receipt of comprehensive CVD risk factor screening: blood pressure, cholesterol, glucose screening, BMI measurement, alcohol consumption and smoking status.

## Methods

### Study design

We used Clinical Practice Research Datalink (CPRD) GOLD and Aurum databases to identify patients with SMI. These databases contain deidentified UK primary care records for patients registered with primary care practices and are broadly representative of the UK population.^19 20^ Our protocol was pre-registered (https://osf.io/czetb/). We investigated annual screening prevalence of six CVD risk factors at the population level, screening patterns at an individual level and patient characteristics associated with receipt of screening.

### Population

We identified patients aged over 18 with a diagnosis of SMI (defined as schizophrenia, bipolar disorder or other non-organic psychosis) in the UK and recorded in CPRD. Entry to the cohort was the latest of SMI diagnosis date, registration at primary care practice or 1 April 2000. Exit was the earliest of death, leaving the primary care practice, 100th birthday or 31 March 2018. Patients did not re-enter the cohort following exit.

Patients were required to be active for at least 1 year between entering and exiting the cohort to allow for screening to be recorded. In line with QOF reporting rules patients had to be registered with their primary care practice for the last 3 months of a financial year to be eligible for screening in that year. In the analysis of factors associated with CVD risk factor screening, we stratified the analysis into three time periods (April 2004–March 2011; April 2011–March 2014; April 2014–March 2018) based on changes to QOF incentivisation (online supplemental table S1). Patients were included in each period if they were eligible for screening for at least two financial years of that period.

### Covariates

We defined the following covariates a priori:

We defined sex, primary care practice and country of primary care practice as recorded in CPRD, and prescription of antipsychotics or mood stabilisers (lithium, sodium valproate or lamotrigine) based on recorded prescriptions issued in primary care. We grouped ethnicity as per the UK 2011 Census.^21^ We defined specific SMI diagnosis as the most recently recorded of schizophrenia, bipolar disorder or other non-organic psychotic illness.

We defined age based on year of birth and categorised as under 40 years or 40 years and older based on year of birth, chosen because cholesterol and blood glucose screening incentivisation was limited to those 40 years and over.^22^ In multinomial logistic regression models, we included age at entry into the cohort as a continuous variable and reported ORs per 10-year increase in age.

We defined presence on other QOF registers which incentivise CVD risk factor screening as having a Read code used in the 2017–2018 QOF incentivisation for any of atrial fibrillation, coronary heart disease, hypertension, peripheral artery disease, stroke or diabetes. We defined those who were exception reported as those with a Read code indicating they had been exception reported from the mental health domain. Exception reporting is the process by which primary care practices may remove patients from the denominator used to calculate incentivised indicators if they are deemed unsuitable for screening, withdrew consent or did not respond. These patients were retained in our analysis.

### Outcomes

We investigated screening of six individual CVD risk factors (cholesterol, blood glucose, blood pressure, BMI, smoking status and alcohol consumption screening) and a composite outcome of all six. For glucose, we included codes for blood glucose or HbA1c tests or values, but excluded urine testing. For cholesterol, we included any screening code or value. For blood pressure, we included screening codes or values for either diastolic or systolic blood pressure. For BMI, we included BMI values, BMI calculated from height and weight and screening codes. For smoking and alcohol status we included any screening code. Further details on the prevalence of CVD risk factors in this cohort are available on the DATAMIND website (https://datamind.org.uk/data/harmonised-data/smi-cohorts/) and code lists used to define the population, covariates and outcomes are available in the Health Data Research UK phenotype library (online supplemental table S2).

In the descriptive analysis our primary outcome was the proportion of patients receiving all six CVD risk factors and each individual risk factor each financial year. As secondary outcomes we investigated the prevalence of ever-recorded CVD risk factor screening and the proportion of patients who ever had all CVD risk factor screening recorded within a 1-month period. A 1-month period was chosen to assess the likelihood that these were recorded as part of a physical health check, with time allowed for recording of results.

In the individual-level analysis of factors associated with CVD risk factor screening, our outcomes were ‘never receiving screening’ and ‘always complete screening’. A patient was considered to have ‘always complete screening’ in a time period if they received screening for all six CVD risk factors in each financial year that they were active in that period.

### Statistical analysis

We calculated the annual prevalence of recorded screening, stratified by the aforementioned covariates. We calculated the proportion of patients receiving screening ever during follow-up (time between a patient entering and exiting the cohort), ever within a 1-month period and for each financial year.

We conducted multinomial logistic regression analyses to assess patient factors associated with receiving ‘always complete’ CVD risk factor screening and receiving no screening in each of the three time periods, compared with receiving irregular screening. ‘Irregular screening’ was defined as any frequency between ‘always complete screening’ and receiving no screening. Irregular screening was chosen as the reference category to allow the comparison of characteristics of those who received no screening to those who received complete screening.

We mutually adjusted for all covariates in the models, with the exception of primary care practice which was used as a clustering term in the calculation of sandwich SEs. We additionally adjusted for time since SMI diagnosis, time since primary care practice registration, year of end of follow-up and total follow-up time. Analysis was performed in R and RStudio and reported in line with the RECORD checklist.^23^

### Missing data

Missing ethnicity was included as a separate category as those with missing ethnicity are different from those with a recorded ethnicity with respect to healthcare access and engagement. For all diagnostic and screening variables, we deemed absence of a code to indicate an absence of diagnosis or screening. We excluded 122 patients who were missing geographical data from the analysis.

### Sensitivity analyses

In a priori sensitivity analysis we limited the population to patients resident in England with available deprivation data (English Index of Multiple Deprivation quintiles). In *post hoc* analysis we limited the model to patients who were active for the whole of the study period due to the strong effect of follow-up time on the completeness of screening.

### Patient and public involvement

Lived experience advisors from the DATAMIND Super Research Advisory Group (https://datamind.org.uk/patients-and-public/the-super-research-advisory-group/) and UCL Mental Health Data Science PPIE group commented on the protocol and provided input into the interpretation of results.

## Findings

We identified 312 471 patients with a diagnostic code for SMI at any time, of whom 216 136 had a diagnosis of SMI before the end of follow-up (time between a patient entering and exiting the cohort), were over the age of 18 years, with at least 1 year of registration and without missing geographical data (online supplemental figure S1). Most patients were resident in England (n=186 880; 86.5%), 1.6% in Northern Ireland, 5.5% in Wales and 6.5% in Scotland. Patients had a median of 4.85 (IQR: 2.43, 9.72) years of follow-up (table 1, online supplemental figure S2).

**Table 1:** Characteristics of the cohort of patients with severe mental illness in CPRD, n=216 136

Characteristic	n (%)/median (**IQR**)
Age at SMI diagnosis (median (IQR))	35 (26, 48)
Age at start of follow-up (median (IQR))	44 (33, 59)
Age at end of follow-up (median (IQR))	52 (39, 68)
Follow-up time* (median (IQR))	4.85 (2.43, 9.72)
Sex, n (%)	
Male	111 655 (51.7)
Female	104 481 (48.3)
Ethnicity, n (%)	
Asian	7679 (3.6)
Black	9979 (4.6)
Mixed	2733 (1.3)
Other	4199 (1.9)
White	110 673 (51.2)
Missing	80 873 (37.4)
Country, n (%)	
England	186 880 (86.5)
Northern Ireland	3405 (1.6)
Scotland	14 010 (6.5)
Wales	11 841 (5.5)
Most recent SMI diagnosis, n (%)	
Schizophrenia	73 753 (34.1)
Bipolar disorder	68 921 (31.9)
Other psychoses	73 462 (34.0)
Ever exception reported, n (%)	59 736 (27.6)
Ever on another CVD QOF register†, n (%)	64 295 (29.8)
Ever prescribed antipsychotics, lithium, sodium valproate or lamotrigine, n (%)	173 669 (80.3)
Died during follow-up*, n (%)	31 210 (14.4)
Age at death (median (IQR))	75.49 (62.44, 84.74)

* Follow-up starts at the latest of 1 April 2000, primary care practice registration, or SMI diagnosis or age 18 a and ends at the earliest of death, leaving the primary care practice, age 100 or last data collection by CPRD.

† Defined as presence on QOF register for atrial fibrillation, coronary heart disease, hypertension, peripheral artery disease, stroke or diabetes.

CPRDClinical Practice Research DatalinkCVD, cardiovascular disease; QOF, Quality and Outcomes FrameworkSMI, severe mental illness

### Population-level analysis of CVD risk factor screening

**Figure 1:**
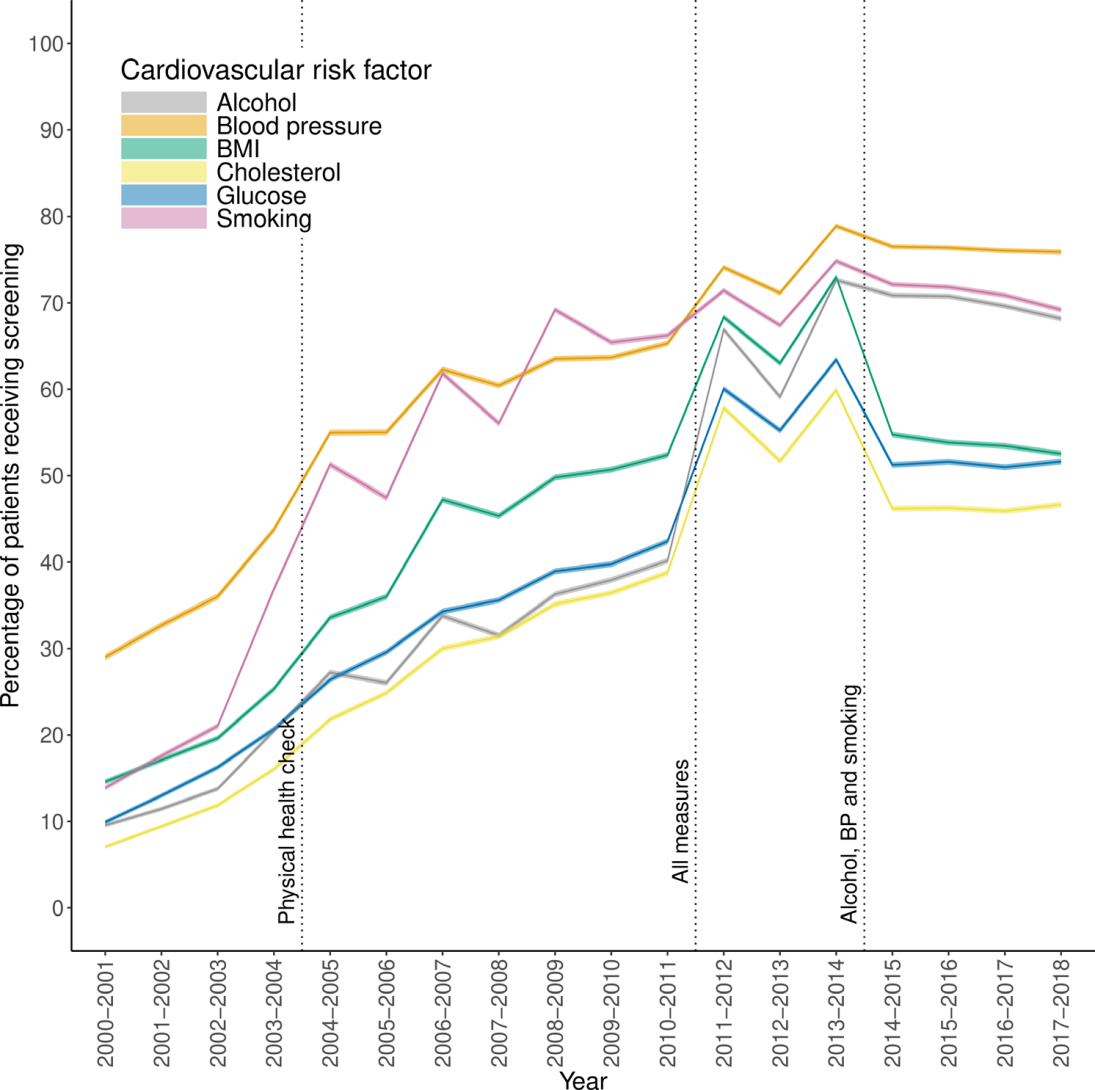
Prevalence of cardiovascular risk factor screening in people with severe mental illness in the primary care setting in the UK by financial year. BMI, body mass index; BP, blood pressure.

The prevalence of smoking and blood pressure screening increased steadily during the study period. In contrast, for alcohol, BMI, cholesterol and glucose screening, the prevalence of screening increased sharply in 2011–2012 following the introduction of incentivisation of individual CVD risk factors. For BMI, cholesterol and glucose screening, the prevalence decreased rapidly from 2013–2014 to 2014–2015, coinciding with the withdrawal of financial incentives (figure 1, online supplemental table S3).

These broad patterns remained when stratified by most patient characteristics (online supplemental figures S3–S11). However, the increase in cholesterol and glucose screening in 2011–2013 was primarily observed in those aged 40 years or older (online supplemental figure S3), the population incentivised at the time. While the prevalence of screening increased for all countries from 2000 to 2014, from 2014 to 2018 (a period of diverging incentivisation across the four nations) the pattern was less consistent (online supplemental figure S4).

Screening for all CVD risk factors was lowest in those with a diagnosis of ‘other psychoses’ (online supplemental figure S5), men (online supplemental figure S6), those not on another QOF register (online supplemental figure S7) and those not on antipsychotics or mood stabilisers (online supplemental figure S8). Screening prevalence for smoking was highest in patients of White or mixed ethnicity, and screening of other CVD risk factors was highest in patients of Asian ethnicity (online supplemental figure S8).

### Individual-level factors associated with receiving ‘always complete’ or no CVD risk factor screening

Almost all patients (93.9%) received screening for at least one CVD risk factor at some point during follow-up. However, only half (54.8%) received screening for all six CVD risk factors at least once (table 2 and online supplemental table S4), and this occurred within a 1-month period for only 34.8% of patients; indicating it is unlikely that screening was not conducted as part of a physical health check. In the period prior to all six CVD risk factors being incentivised (2004–2011), 1.7% of patients received ‘always complete’ screening (ie, all six CVD risk factors each financial year that they were active in 2004–2011). This increased to 14.8% during the period of individual incentivisation (2011–2014) and decreased to 8.3% following that (2014–2018).

**Table 2:** Proportion of patients with severe mental illness ever receiving screening for any of the six cardiovascular disease (CVD) risk factors; and all six CVD risk factors, stratified by covariates, 2000–2018

	n	Any screening measure ever, n (%)	Ever received all 6 measures, n (%)
All	216 136	202 900 (93.9)	118 351 (54.8)
Age at start of follow-up			
<40	85 094	78 660 (92.4)	37 467 (44.0)
40+	131 042	124 240 (94.8)	80 884 (61.7)
Sex			
Male	111 655	103 497 (92.7)	59 466 (53.3)
Female	104 481	99 403 (95.1)	58 885 (56.4)
Ethnicity			
Asian	7679	7533 (98.1)	5457 (71.1)
Black	9979	9697 (97.2)	6366 (63.8)
Mixed	2733	2677 (98.0)	1575 (57.6)
Other	4199	4044 (96.3)	2222 (52.9)
White	110 673	109 203 (98.7)	73 018 (66.0)
Missing	80 873	69 740 (86.2)	29 677 (36.7)
Country			
England	186 880	175 491 (93.9)	101 644 (54.4)
Northern Ireland	3405	3220 (94.6)	2208 (64.8)
Scotland	14 010	12 971 (92.6)	7708 (55.0)
Wales	11 841	11 218 (94.7)	6791 (57.35)
SMI diagnosis			
Schizophrenia	73 753	68 449 (92.8)	42 132 (57.1)
Bipolar disorder	68 921	65 874 (95.6)	40 529 (58.8)
Other psychoses	73 462	68 577 (93.4)	35 690 (48.6)
Ever exception reported			
No	156 376	144 623 (92.5)	82 463 (52.73)
Yes	59 760	58 277 (97.5)	35 888 (60.05)
Ever other QOF**			
No	151 841	139 715 (92.0)	69 972 (46.08)
Yes	64 295	63 185 (98.2)	48 379 (75.2)
Ever prescribed antipsychotics/mood stabilisers			
No	42 467	37 439 (88.2)	15 399 (36.3)
Yes	173 669	165 461 (95.3)	102 952 (59.3)

* Defined as presence on QOF register for atrial fibrillation, coronary heart disease, hypertension, peripheral artery disease, stroke or diabetes.

CVDcardiovascular diseaseQOF, Quality and Outcomes FrameworkSMI, severe mental illness

The odds of receiving no screening in the 2014–2018 and 2011–2014 periods were higher for men, patients of ‘other’ or missing ethnicity (compared with White ethnicity) and those who had been exception reported (ie, deemed unsuitable, withdrew consent or did not respond) from QOF (online supplemental table S5), even after mutual adjustment for covariates (table 3). Conversely, those on other QOF registers which incentivise screening or who had been prescribed antipsychotics or mood stabilisers were less likely to receive no screening in each time period (table 3). In adjusted analyses, compared with patients in England, patients resident in Scotland or Wales were less likely to receive no screening in the 2011–2014 period, but more likely to receive no screening in the 2014–2018 period when both countries reduced incentives in varying ways (table 3, online supplemental table S1).

**Table 3:** Adjusted multinomial logistic regression* for the OR of always receiving complete screening* or receiving no screening compared with irregular screening, among people with severe mental illness, during two time periods

Reference: irregular screening	April 2011–March 2014 (n=85 274)		April 2014–March 2018 (n=94 216)	
	Complete (n=12 616, 14.79%)	None (n=3204, 3.76%)	Complete (n=7771, 8.25%)	None (n=2092, 2.22%)
Age at start of follow-up				
Per 10-year increase	**1.18 (1.17to 1.21)**	1.01 (0.96 to 1.05)	**1.15 (1.14to 1.17)**	**0.86 (0.83to 0.90)**
Sex (ref Female)				
Male	**1.24 (1.19to 1.30)**	**1.31 (1.21to 1.43)**	**1.33 (1.26to 1.41)**	**1.41 (1.27to 1.56)**
Ethnicity (ref White)				
Asian	**1.24 (1.09to 1.41)**	1.00 (0.78 to 1.29)	**1.75 (1.51to 2.04)**	0.88 (0.66 to 1.17)
Black	0.93 (0.82 to 1.06)	**1.31 (1.03to 1.67)**	**1.39 (1.19to 1.62)**	1.23 (0.98 to 1.55)
Mixed	**0.81 (0.67to 0.97)**	1.02 (0.69 to 1.50)	1.04 (0.85 to 1.28)	0.83 (0.54 to 1.28)
Other	**0.77 (0.65to 0.92)**	**2.56 (1.98to 3.31)**	0.83 (0.67 to 1.03)	**1.66 (1.29to 2.15)**
Missing	**0.69 (0.63to 0.76)**	**5.26 (4.34to 6.38)**	**0.70 (0.63to 0.78)**	**2.15 (1.89to 2.44)**
Country (ref England)				
Northern Ireland	1.14 (0.75 to 1.73)	**0.33 (0.22to 0.48)**	**0.60 (0.38to 0.97)**	1.17 (0.78 to 1.74)
Scotland	**1.56 (1.32to 1.86)**	**0.67 (0.53to 0.84)**	1.15 (0.93 to 1.44)	**2.88 (2.43to 3.41)**
Wales	**0.81 (0.67to 0.97)**	**0.48 (0.36to 0.64)**	**0.65 (0.54to 0.79)**	**1.51 (1.21to 1.88)**
SMI diagnosis (ref Bipolar disorder)				
Schizophrenia	**1.27 (1.20to 1.34)**	0.99 (0.89 to 1.10)	**1.30 (1.22to 1.39)**	0.90 (0.79 to 1.03)
Other psychoses	**0.92 (0.88to 0.97)**	1.05 (0.94 to 1.17)	0.96 (0.90 to 1.02)	**1.22 (1.09to 1.37)**
In period variables†				
Exception reported‡	**0.38 (0.36to 0.41)**	**1.26 (1.08to 1.47)**	**0.47 (0.44to 0.51)**	**1.69 (1.51to 1.89)**
Other QOF register§	**1.93 (1.82to 2.04)**	**0.31 (0.28to 0.35)**	**3.21 (2.93to 3.53)**	**0.35 (0.31to 0.39)**
On antipsychotics/mood stabilisers	**1.89 (1.77to 2.01)**	**0.17 (0.15to 0.19)**	**1.95 (1.80to 2.11)**	**0.24 (0.21to 0.26)**
Years since diagnosis	1.00 (1.00 to 1.00)	1.00 (1.00 to 1.00)	1.00 (1.00 to 1.00)	1.00 (1.00 to 1.00)
Years since registration	1.00 (1.00 to 1.00)	1.00 (1.00 to 1.00)	1.00 (1.00 to 1.00)	1.00 (1.00 to 1.00)
Years of follow-up	1.00 (1.00 to 1.00)	1.00 (1.00 to 1.00)	1.00 (1.00 to 1.00)	1.00 (1.00 to 1.00)
Year of end of record in period (ref Final year)¶				
Year 2	**0.00 (00.00to 0.00)**	**0.00 (00.00to 0.00)**	0.28 (0.04 to 2.14)	**4.67 (1.05to 20.71)**
Year 3	NA	NA	**1.35 (1.21to 1.50)**	**2.47 (2.10to 2.90)**

Data presented as OR (95% CI).

Patients could be present in both time periods. The total number of unique patients is 119 976.

Numbers in bold show a higher or lower risk of screening than the reference group.

* Multinomial logistic regression comparing patients receiving no screening or receiving complete screening (all six cardiovascular risk factors for each year that the patient is active in the time period) to those who were irregularly screened, with mutual adjustment for all covariates.

† In period variables measured cross-sectionally, up to the end of the period of interest.

‡ Exception reported from mental health measures.

§ Defined as presence on QOF register for atrial fibrillation, coronary heart disease, hypertension, peripheral artery disease, stroke or diabetes.

¶ Defined as the year a patient ends follow-up. For the 2014–2018 cohort, year two2 is 2015–2016, year 3 is 2016–2017 and the final year is 2017–2018. For the 2011–2014 cohort, year 2 is 2012–2013 and the final year is 2013–2014.

QOF, Quality and Outcomes Frameworkref, reference category; SMI, severe mental illness

In the 2014–2018 period only, older age was associated with lower odds of receiving no screening (OR per 10-year increase in age 0.86; 95% CI 0.83 to 0.90), and compared with bipolar disorder, a diagnosis of ‘other psychoses’ was associated with higher odds of receiving no screening (OR 1.22; 95% CI 1.09 to 1.37; table 3). In the 2011–2014 period only, compared with White ethnicity, Black ethnicity was associated with higher odds of receiving no screening (OR 1.31; 95% CI 1.03 to 1.67).

Men were both more likely to have received no screening, and to have always received complete screening than women in both time periods. Men, older patients, those of Asian ethnicity (compared with those of White ethnicity), with a diagnosis of schizophrenia (compared with bipolar disorder), prescribed antipsychotics or mood stabilisers, on a QOF register that incentivised CVD risk factor and who were never exception reported were more likely to always receive complete screening in all time periods in unadjusted and adjusted models (table 3 and online supplemental tables S6 and S7). Patients of Black (vs White) ethnicity were more likely to always have complete screening in the 2014–2018 period only (OR 1.39; 95% CI 1.19 to 1.62; table 3).

Characteristics associated with receipt of screening differed for individual CVD risk factors (online supplemental tables S4 and S8). For example, in the 2014–2018 period men were more likely to have received regular glucose, cholesterol or smoking screening, but women were more likely to have received regular blood pressure screening. Patients of Black ethnicity were less likely to have received no screening for glucose, cholesterol and BMI, had similar odds of alcohol and blood pressure screening, but were more likely to have received no smoking screening than patients of White ethnicity (online supplemental table S8).

### Sensitivity analysis: investigating the effect of deprivation and follow-up time

Limiting the population to those with available deprivation data or those with complete follow-up for each period did not alter most findings (online supplemental tables S8 and S9). Deprivation was not associated with receiving always complete or no screening, except for in the 2014–2018 period, where those in the most deprived quintile were less likely to have received no screening than those in the least deprived quintile (OR 0.74; 95% CI 0.56 to 0.99; online supplemental table S8).

## Discussion

We found that CVD risk factor screening prevalence varied by risk factor, over time, and by patient characteristics. Our findings suggest that patient characteristics and financial incentivisation influence screening prevalence of individual CVD risk factors, the likelihood of receiving screening for all six CVD risk factors annually and risk of receiving no screening. However, as the patient characteristics associated with increased risk of receiving no screening change over the study periods, it is likely that the groups most at risk of missing screening are dynamic and dependent on which measures are incentivised. Between 2014 and 2018, men, younger patients and those without pre-existing conditions, not prescribed antipsychotics or mood stabilisers, of missing or ‘other’ ethnicity, or with a diagnosis of ‘other psychoses’ had an elevated risk of receiving no screening.

While for those with missing ethnicity, this is likely driven by lack of healthcare contact underpinning both poor ethnicity recording and low levels of screening, for those of younger age or without other physical health conditions it may be driven by additional factors, such as patient and provider perceptions of need.

We found a sharp increase in CVD risk factor screening in 2011, particularly for alcohol, BMI, cholesterol and glucose, and coinciding with the introduction of incentivisation of all six CVD risk factors. The subsequent reduction in prevalence of glucose, cholesterol and BMI screening in 2014 coincides with the removal of these from incentivisation in England and Northern Ireland.^22^ This is in line with previous studies showing a significant reduction in screening for cholesterol and BMI in this period compared with blood pressure in patients with SMI,^18^ and a decrease in target achievement when incentivisation is withdrawn in the general population.^24^ Additionally, we found that incentivisation confers little benefit to those outside of the incentivisation criteria. For example, when cholesterol and glucose screening was incentivised for patients with SMI aged over 40, there was minimal increase in screening in those under 40, so little evidence of halo effects. Likewise, those with ‘other psychoses’ had a lower screening prevalence of all CVD risk factors than patients with schizophrenia or bipolar disorder. The term ‘other psychoses’ covers a range of psychotic diagnoses and symptoms and is broader than the list included under QOF incentivisation. These findings highlight the careful planning needed to ensure those falling outside of incentivisation but still at risk are not marginalised, particularly in those with SMI, who may develop multimorbidity at an early age^1^ and where formal diagnosis may be delayed. Incentivisation has been shown to increase CVD risk factor screening for those with SMI, bringing it in line with that of the general population, or in the case of alcohol screening surpassing the screening prevalence seen in those without SMI.^14 16 17^ While this is encouraging, our results show that screening prevalence differs by patient characteristics and for some patients inequalities still exist. Furthermore, the increased CVD risk profile of those with SMI means that all patients should be receiving CVD risk factor screening, whereas in the general population, those without any risk factors will not be invited to screening. A recent study found that in a population of patients with diabetes, in the 2010–2011 financial year, 94% had HbA1c recorded, 96% had blood pressure recorded, 91% cholesterol, 89% BMI and 83% smoking, far higher than the screening prevalence of those with SMI.^25^ In 2023, the proportion of patients with SMI receiving screening for all six CVD risk factors was incentivised for the first time.^26^ While data collected by NHS England for physical health checks performed in primary or secondary care suggest this has increased the prevalence of screening,^27^our study highlights the need to also consider regularity of screening and how screening varies by patient characteristics.

### Strengths and limitations

The large population size allowed us to stratify results by a range of patient characteristics, while the representative nature of CPRD data makes these results generalisable to the UK for this time period, although with notable differences across the four nations of the UK from 2014. Focusing on longitudinal screening at an individual level, as well as screening prevalence at a population level allowed a better understanding of screening practice over time and the identification of groups of patients who are at risk of receiving no screening. Our results highlight the importance of considering regularity and comprehensiveness of screening at an individual level when evaluating screening interventions, rather than reliance on prevalence of screening at a population level.

We required that patients had at least 1 year of follow-up during the study period to reliably capture screening activity. This may mean that patients who are transiently registered, who are less likely to be screened, are not included in our results. Our analysis was exploratory in nature and while we investigated a range of factors that we hypothesised were associated with receipt of CVD risk factor screening in primary care, it is likely that many of these factors interact to produce distinct risk profiles. Furthermore, we did not investigate outcomes of screening practices and therefore cannot determine the impact that screening has on, for example, stopping smoking, having controlled blood pressure or starting statins, nor on the longer term health of people with SMI. There is a need for further hypothesis-driven studies to identify groups of patients at risk of missed screening, the impact this has on cardiovascular health and into the effectiveness of physical health checks and CVD risk factor screening in this population.

### Clinical implications

People with SMI are at risk of physical health conditions beyond CVD^1^ and of avoidable physical health hospitalisations.^28^ Current NHS guidance describes the incentivised CVD risk factors as core to the physical health check in primary care, but recommends a more comprehensive annual review of physical health.^29^ However, our findings suggest that CVD risk factors may be captured opportunistically. While opportunistic screening results in higher screening prevalence, screening is only a first step. It is important that patients also receive a comprehensive clinical review of physical health so that coordinated actions can be put in place to manage CVD risk, and diagnose and treat conditions beyond CVD. Clinicians should also be aware that while reported screening prevalence of individual CVD risk factors may be high, few patients have complete screening of all measures, and there are inequalities in who receives these.

While physical health screening is embedded in primary care, further consideration is warranted as to the provision of physical health checks in mental health services to complement those in primary care and to provide services to those not regularly in contact with their primary care provider. However, this approach is reliant on improved coordination between physical and mental healthcare providers to avoid duplication for both patient and practitioner, and to ensure transfer of important patient information.

## Conclusions

The low proportion of people receiving regular CVD risk factor screening suggests that people with SMI are not reliably receiving regular comprehensive physical health checks in primary care. Further consideration is warranted as to how incentivisation and other schemes could improve the regularity and comprehensiveness of screening, rather than just the annual screening prevalence, and whether provision of physical health checks in both physical and mental health services may improve uptake. Hypothesis-driven work is required to identify groups of patients most at risk of not receiving CVD risk factor screening so that targeted interventions can be developed, with consideration of these groups in the planning and implementation of future incentivisation and other improvement schemes.

## supplementary material

10.1136/bmjment-2024-301409 online supplemental file 1

## Data Availability

Data may be obtained from a third party and are not publicly available.
