# Supplementary material for: Prevalence and patient characteristics associated with cardiovascular disease risk factor screening in UK primary care for people with severe mental illness: an electronic healthcare record study
Source: BMJ Ment Health. 2025 Jan 15;28(1):e301409. doi: 10.1136/bmjment-2024-301409 (PMC11751913; doi:10.1136/bmjment-2024-301409)
Supplement: online supplemental file 1 [file bmjment-28-1-s001.pdf]

Supplementary material

Table S1: QOF timeline illustrating which physical health risk factors were incentivised during the study period (2004-2018) and after (2019-2024)

Year	Mental health indicators in England	Four nations approach	Smoking indicator	Screening frequency	Other changes
2004/2005	Single indicator for physical health screening	All four nations apply the same rules		15 months	
2005/2006					
2006/2007			Smoking indicator added, but does not include SMI		1st major revision and exception reporting guidance
2007/2008					
2008/2009			Smoking indicator includes SMI		
2009/2010					
2010/2011					
2011/2012	Individual indicators for alcohol consumption, blood pressure, BMI, cholesterol and glucose monitoring and cervical screening				
2012/2013					
2013/2014				12 months (England only)	
2014/2015	Individual indicators for alcohol consumption, blood pressure and cervical screening	Scotland, Wales and Northern Ireland apply differing rules*			
2015/2016					
2016/2017		Scotland abolish QOF			
2017/2018					
2018/2019					Changed from Read codes to SNOMED
2019/2020		Wales abolish QOF			Physical health check data collected as part of Long Term Plan in England
2020/2021	Individual indicators for blood pressure and BMI				Changes due to COVID
2021/2022					Changes due to COVID
2022/2023	Individual indicators for alcohol consumption, blood pressure, BMI, cholesterol and glucose monitoring				
2023/2024	Individual indicators for alcohol consumption, blood pressure, BMI, cholesterol and glucose monitoring plus incentivisation for all five and smoking				

Taken from [22] and [31]. BMI: Body mass index; QOF: Quality and Outcomes Framework

*From 2014: In Wales QOF incentivised glucose, blood pressure and BMI screening in a 15-month period as one indicator and alcohol, smoking and cervical screening in a 15-month period as individual indicators [32 33]. In Scotland QOF incentivised screening of all six cardiovascular risk factors as individual indicators in a 15-month period [34]. In Northern Ireland, QOF incentivised blood pressure, smoking and cervical screening in a 15 month period [35].

Table S2: Code lists for outcomes, population and covariates

Condition	Link to code list
Severe mental illness	https://phenotypes.healthdatagateway.org/phenotypes/PH1649/version/3407/detail/
Glucose	https://phenotypes.healthdatagateway.org/phenotypes/PH1656/version/3422/detail/
Cholesterol	https://phenotypes.healthdatagateway.org/phenotypes/PH1655/version/3421/detail/
Blood pressure	https://phenotypes.healthdatagateway.org/phenotypes/PH1654/version/3420/detail/
BMI	https://phenotypes.healthdatagateway.org/phenotypes/PH1652/version/3404/detail/
Smoking	https://phenotypes.healthdatagateway.org/phenotypes/PH1651/version/3408/detail/
Alcohol	https://phenotypes.healthdatagateway.org/phenotypes/PH1678/version/3543/detail/
Antipsychotics/mood stabilisers	https://phenotypes.healthdatagateway.org/phenotypes/PH1650/version/3406/detail/
Exception reporting	https://phenotypes.healthdatagateway.org/phenotypes/PH1679/version/3544/detail/

Table S3: Annual prevalence of individual cardiovascular risk factor screening in patients with severe mental illness by financial year, n=216,136

	**Cholesterol**		**Glucose**		**Blood pressure**		**BMI**		**Smoking**		**Alcohol**	
**Year, (total eligible)**	Number screened	Screening prevalence (95%CI)	Number screened	Screening prevalence (95%CI)	Number screened	Screening prevalence (95%CI)	Number screened	Screening prevalence (95%CI)	Number screened	Screening prevalence (95%CI)	Number screened	Screening prevalence (95%CI)
**2000-2001 (57,814)**	4,083	7.1 (6.9-7.3)	5,731	9.9 (9.7-10.1)	16,771	29.0 (28.64-29.4)	8,435	14.6 (14.3-14.9)	8,025	13.9 (13.6-14.2)	5,518	9.5 (9.3-9.8)
**2001-2002 (61,418)**	5,783	9.4 (9.2-9.6)	7,978	13.0 (12.7-13.3)	20,077	32.7 (32.3-33.1)	10,503	17.1 (16.8-17.4)	10,795	17.6 (17.3-17.9)	7,036	11.5 (11.2-11.7)
**2002-2003 (65,266)**	7,743	11.9 (11.6-12.1)	10,616	16.3 (16.0-16.6)	23,507	36.0 (35.7-36.4)	12,811	19.6 (19.3-19.9)	13,735	21.0 (20.7-21.4)	8,988	13.8 (13.5-14.0)
**2003-2004 (69,276)**	11,117	16.1 (15.8-16.3)	14,333	20.7 (20.4-21.0)	30,327	43.8 (43.4-44.2)	17,552	25.3 (25.0-25.7)	25,550	36.9 (36.5-37.2)	14,193	20.5 (20.2-20.8)
**2004-2005 (72,827)**	15,871	21.8 (21.5-22.1)	19,226	26.4 (26.1-26.7)	40,029	55.0 (54.6-55.3)	24,466	33.6 (33.3-33.9)	37,349	51.3 (50.9-51.7)	19,821	27.2 (26.9-27.5)
**2005-2006 (75,007)**	18,658	24.9 (24.6-25.2)	22,190	29.6 (29.3-29.9)	41,262	55.0 (54.7-55.4)	26,997	36.0 (35.7-36.3)	35,584	47.4 (47.1-47.8)	19,523	26.0 (25.7-26.3)
**2006-2007 (76,951)**	23,092	30.0 (29.7-30.3)	26,385	34.3 (34.0-34.6)	47,926	62.3 (61.9-62.6)	36,339	47.2 (46.9-47.6)	47,575	61.8 (61.5-62.2)	26,005	33.8 (33.5-34.1)
**2007-2008 (79,059)**	24,792	31.4 (31.0-31.7)	28,143	35.6 (35.3-35.9)	47,779	60.4 (60.1-60.8)	35,842	45.3 (45.0-45.7)	44,313	56.1 (55.7-56.4)	24,921	31.5 (31.2-31.9)
**2008-2009 (80,372)**	28,235	35.1 (34.8-35.5)	31,297	38.9 (38.6-39.3)	51,040	63.5 (63.2-63.8)	40,017	49.8 (49.4-50.1)	55,614	69.2 (68.9-69.5)	29,154	36.3 (35.9-36.6)
**2009-2010 (82,242)**	29,961	36.4 (36.1-36.8)	32,697	39.8 (39.4-40.1)	52,356	63.7 (63.3-64.0)	41,694	50.7 (50.4-51.0)	53,808	65.4 (65.1-65.8)	31,197	37.9 (37.6-38.3)
**2010-2011 (83,749)**	32,474	38.8 (38.5-39.1)	35,506	42.4 (42.1-42.7)	54,693	65.3 (65.0-65.6)	43,884	52.4 (52.1-52.7)	55,460	66.2 (65.9-66.5)	33,623	40.2 (39.8-40.5)
**2011-2012 (85,592)**	49,507	57.8 (57.5-58.2)	51,386	60.0 (59.7-60.4)	63,421	74.1 (73.8-74.4)	58,492	68.3 (68.0-68.7)	61,127	71.4 (71.1-71.7)	57,266	66.9 (66.6-67.2)
**2012-2013 (86,870)**	44,904	51.7 (51.4-52.0)	47,984	55.2 (54.9-55.6)	61,797	71.1 (70.8-71.4)	54,729	63.0 (62.7-63.3)	58,538	67.4 (67.1-67.7)	51,355	59.1 (58.8-59.4)
**2013-2014 (85,921)**	51,459	59.9 (59.6-60.2)	54,479	63.4 (63.1-63.7)	67,781	78.9 (78.6-79.2)	62,698	73.0 (72.7-73.3)	64,300	74.8 (74.5-75.1)	62,395	72.6 (72.3-72.9)
**2014-2015 (86,674)**	40,036	46.2 (45.9-46.5)	44,409	51.2 (50.9-51.6)	66,290	76.5 (76.2-76.8)	47,447	54.7 (54.4-55.1)	62,501	72.1 (71.8-72.4)	61,415	70.9 (70.6-71.2)

**2015-2016 (86482)**	40,002	46.3 (45.9-46.6)	44,632	51.6 (51.3-51.9)	66,034	76.4 (76.1-76.6)	46,546	53.8 (53.5-54.2)	62,107	71.8 (71.5-72.1)	61,194	70.8 (70.5-71.1)
**2016-2017 (86895)**	39,885	45.9 (45.6-46.2)	44,299	51.0 (50.7-51.3)	66,059	76.0 (75.7-76.3)	46,440	53.4 (53.1-53.8)	61,581	70.9 (70.6-71.2)	60,504	69.6 (69.3-69.9)
**2017-2018 (80419)**	37,515	46.7 (46.3-47.0)	41,525	51.6 (51.3-52.0)	61,024	75.9 (75.6-76.2)	42,254	52.5 (52.2-52.9)	55,644	69.2 (68.9-69.5)	54,818	68.2 (67.8-68.5)

95%CI: 95% confidence intervals; BMI: body mass index

Table S4: Proportion of patients with severe mental illness ever receiving screening for: each cardiovascular disease (CVD) risk factor; any of the six CVD risk factors; and all six CVD risk factors, stratified by covariates, 2000-2018

		**n**	**Cholesterol, n(%)**	**Glucose, n(%)**	**Blood pressure, n(%)**	**BMI, n(%)**	**Smoking, n(%)**	**Alcohol, n(%)**	**Any screening measure ever, n(%)**	**Ever received all 6 measures, n(%)**
**All**		216,136	136,897 (63.3)	151,652 (70.2)	189,844 (87.8)	168,378 (77.9)	190,967 (88.4)	172,441 (79.8)	202,900 (93.9)	118,351 (54.8)
**Age at start of follow-up**	Under 40	85,094	42,548 (50.0)	49,781 (58.5)	70,464 (82.8)	65,073 (76.5)	75,353 (88.55)	67,778 (79.7)	78,660 (92.4)	37,467 (44.0)
	40+	131,042	94,349 (72.0)	101,871 (77.7)	119,380 (91.1)	103,305 (78.8)	115,614 (88.2)	104,663 (79.9)	124,240 (94.8)	80,884 (61.7)
**Sex**	Male	111,655	68,285 (61.2)	73,664 (66.0)	94,744 (84.6)	85,708 (76.8)	98,423 (88.1)	89,405 (80.1)	103,497 (92.7)	59,466 (53.3)
	Female	104,481	68,612 (65.7)	77,988 (74.6)	95,100 (91.0)	82,670 (79.1)	92,544 (88.6)	83,036 (79.5)	99,403 (95.1)	58,885 (56.4)
**Ethnicity**	Asian	7,679	5,896 (76.8)	6,234 (81.2)	7,192 (93.7)	6,886 (89.7)	7,317 (95.3)	7,079 (92.2)	7,533 (98.1)	5,457 (71.1)
	Black	9,979	6,966 (69.8)	7,386 (74.0)	9,189 (92.1)	8,684 (87.0)	9,410 (94.3)	8,933 (89.5)	9,697 (97.2)	6,366 (63.8)
	Mixed	2,733	1,755 (64.2)	1,885 (69.0)	2,472 (90.5)	2,344 (85.8)	2,610 (95.5)	2,455 (89.8)	2,677 (98.0)	1,575 (57.6)
	Other	4,199	2,652 (63.2)	2,969 (70.7)	3,710 (88.4)	3,264 (77.7)	3,860 (91.9)	3,382 (80.5)	4044 (96.3)	2,222 (52.9)
	White	110,673	80,734 (72.9)	86,716 (78.4)	103,788 (93.8)	97397 (88.0)	106,716 (96.4)	100,784 (91.1)	109,203 (98.7)	73,018 (66.0)
	Missing	80,873	38,894 (48.1)	46,462 (57.5)	63,493 (78.5)	49,803 (61.6)	61,054 (75.5)	49,808 (61.6)	69,740 (86.2)	29,677 (36.7)
**Country**	England	186,880	117,627 (62.9)	130,688 (69.9)	164,352 (87.9)	145,296 (77.7)	165,068 (88.3)	149,262 (79.9)	175,491 (93.9)	101,644(54.4)
	Northern Ireland	3,405	2,546 (74.8)	2,716 (79.8)	3,069 (90.1)	2,816 (82.7)	3,053 (89.7)	2,797 (82.1)	3,220 (94.6)	2,208 (64.8)
	Scotland	14,010	8,975 (64.1)	9,422 (67.3)	11,994 (85.6)	10,813 (77.2)	122,27 (87.3)	10,835 (77.3)	12,971 (92.6)	7,708 (55.0)
	Wales	11,841	7,749 (65.4)	8,826 (74.5)	10,429 (88.1)	9,453 (79.8)	10,619 (89.7)	9,547 (80.6)	11,218 (94.7)	6,791 (57.35)
**SMI diagnosis**	Schizophrenia	73,753	47,869 (64.9)	51,813 (70.3)	64,368 (87.3)	57,927 (78.5)	64,902 (88.0)	58,788 (79.7)	68,449 (92.8)	42,132 (57.1)
	Bipolar disorder	68,921	46,587 (67.6)	51,431 (74.6)	62,488 (90.7)	56,195 (81.5)	62,394 (90.5)	56,951 (82.6)	65,874 (95.6)	40,529 (58.8)
	Other psychoses	73,462	42,441 (57.7)	48,408 (65.9)	62,988 (85.7)	54,256 (73.9)	63,671 (86.7)	56,702 (77.2)	68,577 (93.4)	35,690 (48.6)

**Ever exception reported**	No	156,376	96,387 (61.6)	107,467 (68.7)	135,588 (86.7)	118,881 (76.0)	134,324 (85.9)	120,774 (77.2)	144,623 (92.5)	82,463 (52.73)
	Yes	59,760	40528 (67.8)	44203 (74.0)	54277 (90.8)	49,497 (82.8)	56667 (94.8)	51691 (86.5)	58,277 (97.5)	35,888 (60.05)
**Ever other QOF [a]**	No	151,841	81,417 (53.6)	94,124 (62.0)	127,397 (83.9)	113,031 (74.4)	131,518 (86.6)	117,208 (77.2)	139,715 (92.0)	69,972 (46.08)
	Yes	64,295	55,480 (86.3)	57,528 (89.5)	62,447 (97.1)	55,347 (86.1)	59,449 (92.5)	55,233 (85.9)	63,185 (98.2)	48,379 (75.2)
**Ever prescribed antipsychotics/mood stabilisers**	No	42,467	19,178 (45.2)	22,071 (52.0)	33,028 (77.8)	28,426 (66.9)	34,662 (81.6)	29,940 (70.5)	37,439 (88.2)	15,399 (36.3)
	Yes	173,669	117,719 (67.8)	129,581 (74.6)	156,816 (90.3)	139,952 (80.6)	156,305 (90.0)	142,501 (82.1)	165,461 (95.3)	102,952 (59.3)

SMI: Severe Mental Illness; BMI: Body mass index; QOF: Quality and Outcomes Framework

a: Defined as presence on QOF register for atrial fibrillation, coronary heart disease, hypertension, peripheral artery disease, stroke or diabetes.

Table S5: Unadjusted multinomial logistic regression for the odds ratio of always receiving complete screening[a] or receiving no screening compared to irregular screening, among people with severe mental illness, during two time periods

		April 2011 – March 2014 (n=85,274)		April 2014 – March 2018 (n=94,216)	
**Reference: Irregular screening**		**Complete (n=12,616, 14.79%)**	**None (n=3,204, 3.76%)**	**Complete (n=7,771, 8.25%)**	**None (n=2,092, 2.22%)**
		**OR (95% CI)**	**OR (95% CI)**	**OR (95% CI)**	**OR (95% CI)**
**Age at start of follow-up**	Per 10-year increase	**1.26 (1.24-1.27)**	1.00 (0.95-1.06)	**1.27 (1.24-1.28)**	**0.82 (0.79-0.94)**
**Sex (ref female)**	Male	1.03 (0.98-1.07)	**1.58 (1.46-1.72)**	**1.09 (1.04-1.15)**	**1.93 (1.75-2.12)**
**Ethnicity (ref White)**	Asian	**1.13 (1.00-1.28)**	1.02 (0.81-1.30)	**1.54 (1.34-1.78)**	0.82 (0.62-1.09)
	Black	**0.81 (0.71-0.92)**	**1.60 (1.27-2.02)**	**1.18 (1.01-1.37)**	**1.35 (1.08-1.68)**
	Mixed	**0.65 (0.53-0.78)**	1.24 (0.85-1.80)	0.83 (0.67-1.01)	0.89 (0.58-1.36)
	Other	**0.68 (0.57-0.81)**	**2.43 (1.88-3.13)**	**0.73 (0.58-0.93)**	**2.12 (1.61-2.79)**
	Missing	**0.73 (0.66-0.80)**	**4.76 (3.92-5.78)**	**0.68 (0.61-0.75)**	**2.62 (2.33-2.95)**
**Country (ref England)**	Northern Ireland	1.04 (0.69-1.57)	**0.55 (0.37-0.79)**	**0.58 (0.35-0.94)**	1.31 (0.90-1.90)
	Scotland	**1.36 (1.17-1.59)**	0.85 (0.68-1.07)	1.06 (0.86-1.30)	**3.02 (2.59-3.52)**
	Wales	**0.66 (0.55-0.79)**	0.80 (0.61-1.58)	**0.52 (0.43-0.65)**	**1.95 (1.57-2.42)**
**SMI diagnosis (ref bipolar disorder)**	Schizophrenia	**1.26 (1.20-1.32)**	**1.31 (1.19-1.45)**	**1.38 (1.30-1.48)**	**1.17 (1.04-1.32)**
	Other psychoses	**0.83 (0.78-0.87)**	**1.44 (1.27-1.63)**	**0.87 (0.82-0.93)**	**1.82 (1.63-2.03)**
**In period variables[d]**	Exception reported[b]	**0.37 (0.35-0.39)**	**1.46 (1.19-1.80)**	**0.45 (0.42-0.48)**	**2.14 (1.92-2.38)**
	Other QOF register[c]	**2.30 (2.17-2.42)**	**0.28 (0.25-0.31)**	**3.71 (3.39-4.07)**	**0.28 (0.52-0.30)**
	On antipsychotics/mood stabilisers	**2.05 (1.93-2.18)**	**0.14 (0.13-0.16)**	**2.14 (1.97-2.32)**	**0.21 (0.19-0.23)**
	Years since diagnosis	1.00 (1.00-1.00)	1.00 (1.00-1.00)	1.00 (1.00-1.00)	1.00 (1.00-1.00)
	Years since registration	1.00 (1.00-1.00)	1.00 (1.00-1.00)	1.00 (1.00-1.00)	1.00 (1.00-1.00)
	Years of follow-up	1.00 (1.00-1.00)	0.998 (0.998-0.999)	1.00 (1.00-1.00)	0.999 (0.999-1.00)
**Year of end of record in period (ref final year)[e]**	Year 2	**0.00 (00.00-0.00)**	**0.00 (00.00-0.00)**	0.43 (0.57-3.15)	**5.07 (1.52-16.87)**
	Year 3	NA	NA	**1.66 (1.51-1.83)**	**2.50 (2.19-2.86)**

OR: Odds ratio; 95% CI: 95% confidence interval; ref: Reference category; SMI: Severe Mental Illness; QOF: Quality and Outcomes Framework

a: Complete screening was defined as screening of all six cardiovascular risk factors for each year that the patient is active
b: Exception reported from mental health measures

c: Defined as presence on QOF register for atrial fibrillation, coronary heart disease, hypertension, peripheral artery disease, stroke or diabetes.
d: In period variables measured cross-sectionally, up to the end of the period of interest
e: Defined as the year a patient ends follow-up. For the 2014-2018 cohort, year two is 2015-2016, year 3 is 2016-2017 and the final year is 2017-2018. For the 2011-2014 cohort year 2 is 2012-2013 and the final year is 2013-2014.
Note: Patients could be present in both time periods. The total number of unique patients is 119,976

Table S6: Multinomial logistic regression for the odds ratios of always receiving complete screening or receiving no screening compared to irregular screening in patients with severe mental illness for the period of April 2004 to March 2011, n=106,747

**Reference: Irregular screening**		**Complete (n=1,770, 1.66%)**	**None (n=5,795, 5.43%)**
		**OR (95% CI)**	**OR (95% CI)**
**Age at start of follow-up**	Per 10-year increase	**1.23 (1.20-1.27)**	**0.95 (0.91-0.98)**
**Sex (ref female)**	Male	**1.38 (1.24-1.53)**	**1.36 (1.26-1.46)**
**Ethnicity (ref White)**	Asian	**2.36 (1.88-2.97)**	1.23 (0.97-1.56)
	Black	**1.54 (1.19-1.99)**	**1.50 (1.23-1.84)**
	Mixed	**1.62 (1.07-2.44)**	1.10 (0.75-1.61)
	Other	**0.55 (0.34-0.88)**	**2.71 (2.15-3.42)**
	Missing	**0.57 (0.48-0.67)**	**4.96 (4.16-5.90)**
**Country (ref England)**	Northern Ireland	0.70 (0.32-1.51)	**0.41 (0.30-0.57)**
	Scotland	1.11 (0.82-1.50)	0.55 (0.46-0.66)
	Wales	1.03 (0.75-1.40)	0.47 (0.37-0.59)
**SMI diagnosis (ref bipolar)**	Schizophrenia	**1.22 (1.08-1.37)**	**1.25 (1.14-1.37)**
	Other psychoses	0.89 (0.78-1.01)	**1.16 (1.05-1.29)**
**In period variables**	Exception reported	**0.55 (0.47-0.65)**	0.98 (0.80-1.19)
	Other QOF register[a]	**8.37 (6.74-10.41)**	**0.31 (0.28-0.33)**
	On antipsychotics/mood stabilisers	**1.29 (1.14-1.46)**	**0.16 (0.14-0.19)**
	Years since diagnosis	1.00 (1.00-1.00)	1.00 (1.00-1.00)
	Years since registration	1.00 (1.00-1.00)	1.00 (1.00-1.00)
	Years of follow-up	1.00 (1.00-1.00)	1.00 (1.00-1.00)
**Year of end of record in period (ref 2010-2011)**	2005-2006	**5.15 (1.30-20.41)**	**4.78 (1.13-20.22)**
	2006-2007	1.05 (0.85-1.30)	**4.91 (4.13-5.84)**
	2007-2008	0.90 (0.73-1.11)	**2.24 (1.89-2.66)**
	2008-2009	0.86 (0.69-1.07)	**2.43 (1.29-4.58)**
	2009-2010	**0.76 (0.61-0.94)**	**1.26 (1.04-1.53)**

OR: Odds ratio; 95% CI: 95% confidence interval; ref: Reference category; SMI: Severe Mental Illness; QOF: Quality and Outcomes Framework

a: Defined as presence on QOF register for atrial fibrillation, coronary heart disease, hypertension, peripheral artery disease, stroke or diabetes.

Table S7: Multinomial logistic regression for the odds ratios of always receiving complete screening or receiving no screening compared to irregular screening for individual cardiovascular risk factors in people with severe mental illness, April 2011-March 2018, n=119,976

**Reference: Irregular screening**			**April 2011 – March 2014 (n=85,274)**		**April 2014 – March 2018 (n=94,216)**	
			**Complete (n=12,616, 14.79%) OR (95% CI)**	**None (n=3,204, 3.76%) OR (95% CI)**	**Complete (n=12,616, 14.79%) OR (95% CI)**	**None (n=3,204, 3.76%) OR (95% CI)**
**Blood pressure**	**Age at start of follow-up**	Per 10-year increase	1.29 (1.27-1.31)	0.92 (0.90-0.96)	1.27 (1.26-1.29)	0.84 (0.83-0.87)
	**Sex (ref female)**	Male	0.90 (0.87-0.93)	1.47 (1.39-1.56)	0.87 (0.84-0.89)	1.51 (1.41-1.60)
	**Ethnicity (ref white)**	Asian	1.34 (1.23-1.47)	0.86 (0.74-1.00)	1.40 (1.27-1.54)	0.85 (0.72-1.00)
		Black	1.27 (1.17-1.38)	0.97 (0.83-1.15)	1.20 (1.10-1.30)	1.06 (0.93-1.21)
		Mixed	1.04 (0.90-1.21)	1.00 (0.80-1.24)	1.04 (0.92-1.18)	1.01 (0.80-1.27)
		Other	0.82 (0.73-0.92)	1.55 (1.31-1.84)	0.95 (0.85-1.07)	1.27 (1.06-1.53)
		Missing	0.74 (0.69-0.78)	2.62 (2.32-2.96)	0.79 (0.75-0.84)	1.53 (1.42-1.66)
	**Country (ref England)**	NI	0.89 (0.74-1.07)	0.45 (0.37-0.55)	0.44 (0.34-0.56)	0.69 (0.52-0.90)
		Scotland	1.05 (0.96-1.16)	0.90 (0.79-1.03)	0.39 (0.34-0.44)	1.87 (1.65-2.12)
		Wales	0.88 (0.80-0.97)	0.64 (0.54-0.76)	0.43 (0.39-0.47)	1.21 (1.04-1.41)
	**SMI diagnosis (ref bipolar disorder)**	Schizophrenia	1.04 (1.00-1.09)	1.03 (0.96-1.11)	0.99 (0.95-1.04)	0.99 (0.91-1.07)
		Other psychoses	0.89 (0.86-0.93)	1.17 (1.09-1.26)	0.89 (0.85-0.92)	1.18 (1.10-1.27)
	**In period variables**	**Exception reported**	0.42 (0.40-0.43)	1.69 (1.54-1.86)	0.37 (0.36-0.39)	1.60 (1.51-1.71)
		**Other QOF register[a]**	2.13 (2.05-2.21)	0.58 (0.54-0.62)	2.01 (1.94-2.08)	0.59 (0.55-0.62)
		**On antipsychotics/mood stabilisers**	1.73 (1.65-1.81)	0.32 (0.30-0.35)	2.07 (1.98-2.16)	0.40 (0.38-0.43)
		**Time since diagnosis**	1.00 (1.00-1.00)	1.00 (1.00-1.00)	1.00 (1.00-1.00)	1.00 (1.00-1.00)
		**Time since registration**	1.00 (1.00-1.00)	1.00 (1.00-1.00)	1.00 (1.00-1.00)	1.00 (1.00-1.00)
		**Years of follow-up**	1.00 (1.00-1.00)	1.00 (1.00-1.00)	1.00 (1.00-1.00)	1.00 (1.00-1.00)
		Year 2	0.66 (0.17-2.52)	0.00 (0.00-0.00)	1.01 (0.46-2.19)	2.78 (0.93-8.33)

Reference: Irregular screening			April 2011 – March 2014 (n=85,274)		April 2014 – March 2018 (n=94,216)	
			Complete (n=12,616, 14.79%)<br>OR (95% CI)	None (n=3,204, 3.76%)<br>OR (95% CI)	Complete (n=12,616, 14.79%)<br>OR (95% CI)	None (n=3,204, 3.76%)<br>OR (95% CI)
	**Year of end of follow-up in period (ref final year)**	Year 3	NA	NA	1.52 (1.38-1.66)	2.27 (2.06-2.51)
**Glucose**	**Age at start of follow-up**	**Per 10-year increase**	1.28 (1.27-1.29)	0.73 (0.71-0.75)	1.21 (1.20-1.22)	0.80 (0.79-0.82)
	**Sex (ref female)**	Male	1.08 (1.05-1.12)	1.19 (1.14-1.23)	1.12 (1.08-1.16)	1.25 (1.21-1.30)
	**Ethnicity (ref white)**	Asian	1.41 (1.29-1.54)	0.80 (0.72-0.89)	1.85 (1.71-2.01)	0.67 (0.60-0.74)
		Black	1.18 (1.08-1.28)	0.97 (0.88-1.07)	1.45 (1.33-1.57)	0.89 (0.82-0.97)
		Mixed	1.02 (0.88-1.18)	1.16 (1.00-1.36)	1.16 (1.00-1.35)	1.04 (0.91-1.19)
		Other	0.89 (0.78-1.01)	1.48 (1.29-1.70)	1.04 (0.90-1.20)	1.13 (0.99-1.28)
		Missing	0.83 (0.78-0.88)	1.84 (1.69-2.01)	0.84 (0.79-0.89)	1.41 (1.33-1.49)
	**Country (ref England)**	NI	1.20 (0.91-1.60)	0.53 (0.43-0.66)	0.98 (0.80-1.21)	0.80 (0.66-0.98)
		Scotland	1.20 (1.07-1.34)	0.94 (0.83-1.07)	1.20 (1.06-1.36)	1.05 (0.95-1.16)
		Wales	0.94 (0.84-1.05)	0.75 (0.67-0.85)	1.24 (1.13-1.36)	0.65 (0.59-0.71)
	**SMI diagnosis (ref bipolar disorder)**	Schizophrenia	1.06 (1.02-1.11)	1.02 (0.97-1.08)	1.17 (1.11-1.22)	1.07 (1.02-1.12)
		Other psychoses	0.88 (0.84-0.91)	1.23 (1.17-1.30)	0.95 (0.91-1.00)	1.26 (1.21-1.32)
	**In period variables**	**Exception reported**	0.46 (0.44-0.48)	1.76 (1.66-1.87)	0.61 (0.58-0.64)	1.78 (1.70-1.86)
		**Other QOF register[a]**	1.77 (1.70-1.85)	0.63 (0.60-0.66)	2.32 (2.21-2.44)	0.66 (0.64-0.69)
		**On antipsychotics/mood stabilisers**	1.90 (1.80-1.99)	0.38 (0.36-0.40)	1.63 (1.55-1.72)	0.45 (0.43-0.47)
		**Time since diagnosis**	1.00 (1.00-1.00)	1.00 (1.00-1.00)	1.00 (1.00-1.00)	1.00 (1.00-1.00)
		**Time since registration**	1.00 (1.00-1.00)	1.00 (1.00-1.00)	1.00 (1.00-1.00)	1.00 (1.00-1.00)
		**Years of follow-up**	1.00 (1.00-1.00)	1.00 (1.00-1.00)	1.00 (1.00-1.00)	1.00 (1.00-1.00)

Reference: Irregular screening			April 2011 – March 2014 (n=85,274)		April 2014 – March 2018 (n=94,216)	
			**Complete (n=12,616, 14.79%)<br>OR (95% CI)**	**None (n=3,204, 3.76%)<br>OR (95% CI)**	**Complete (n=12,616, 14.79%)<br>OR (95% CI)**	**None (n=3,204, 3.76%)<br>OR (95% CI)**
	**Year of end of follow-up in period (ref final year)**	Year 2	0.41 (0.09-1.93)	0.00 (00.00-0.00)	0.90 (0.29-2.80)	3.08 (1.29-7.39)
		Year 3	NA	NA	1.56 (1.45-1.69)	1.64 (1.52-1.78)
**Cholesterol**	**Age at start of follow-up**	Per 10-year increase	1.23 (1.22-1.24)	0.68 (0.66-0.69)	1.16 (1.15-1.18)	0.78 (0.77-0.80)
	**Sex (ref female)**	Male	1.16 (1.12-1.20)	0.99 (0.95-1.03)	1.21 (1.16-1.25)	0.97 (0.94-1.01)
	**Ethnicity (ref white)**	Asian	1.34 (1.22-1.48)	0.80 (0.72-0.89)	1.72 (1.58-1.88)	0.63 (0.57-0.70)
		Black	1.08 (0.98-1.18)	0.90 (0.82-1.00)	1.42 (1.30-1.54)	0.82 (0.76-0.89)
		Mixed	0.98 (0.85-1.14)	1.10 (0.94-1.28)	1.22 (1.04-1.43)	1.05 (0.92-1.19)
		Other	0.87 (0.77-0.99)	1.49 (1.31-1.69)	0.94 (0.80-1.09)	1.18 (1.05-1.33)
		Missing	0.78 (0.73-0.83)	1.80 (1.66-1.96)	0.85 (0.79-0.92)	1.51 (1.42-1.59)
	**Country (ref England)**	Northern Ireland	1.14 (0.85-1.52)	0.57 (0.47-0.69)	0.99 (0.73-1.33)	0.84 (0.69-1.03)
		Scotland	1.32 (1.18-1.47)	0.92 (0.83-1.03)	1.46 (1.24-1.71)	1.08 (0.96-1.21)
		Wales	0.87 (0.78-0.98)	0.77 (0.69-0.87)	0.80 (0.69-0.92)	0.91 (0.81-1.02)
	**SMI diagnosis (ref bipolar disorder)**	Schizophrenia	1.02 (0.98-1.06)	0.95 (0.90-0.99)	1.12 (1.07-1.17)	0.92 (0.88-0.96)
		Other psychoses	0.87 (0.84-0.91)	1.25 (1.19-1.31)	0.95 (0.90-0.99)	1.23 (1.18-1.28)
	**In period variables**	**Exception reported**	0.47 (0.45-0.49)	1.78 (1.69-1.88)	0.61 (0.58-0.64)	1.70 (1.63-1.77)
		**Other QOF register[a]**	1.54 (1.48-1.60)	0.65 (0.62-0.68)	2.15 (2.03-2.27)	0.69 (0.67-0.72)
		**On antipsychotics/mood stabilisers**	1.64 (1.57-1.73)	0.37 (0.35-0.39)	1.46 (1.38-1.55)	0.45 (0.43-0.47)
		**Time since diagnosis**	1.00 (1.00-1.00)	1.00 (1.00-1.00)	1.00 (1.00-1.00)	1.00 (1.00-1.00)
		**Time since registration**	1.00 (1.00-1.00)	1.00 (1.00-1.00)	1.00 (1.00-1.00)	1.00 (1.00-1.00)
		**Years of follow-up**	1.00 (1.00-1.00)	1.00 (1.00-1.00)	1.00 (1.00-1.00)	1.00 (1.00-1.00)
		Year 2	0.34 (0.08-1.49)	0.26 (0.02-2.90)	0.52 (0.13-2.01)	3.22 (1.34-7.78)

Reference: Irregular screening			April 2011 – March 2014 (n=85,274)		April 2014 – March 2018 (n=94,216)	
			Complete (n=12,616, 14.79%)<br>OR (95% CI)	None (n=3,204, 3.76%)<br>OR (95% CI)	Complete (n=12,616, 14.79%)<br>OR (95% CI)	None (n=3,204, 3.76%)<br>OR (95% CI)
	**Year of end of follow-up in period (ref final year)**	Year 3	NA	NA	1.50 (1.38-1.63)	1.73 (1.60-1.88)
**BMI**	**Age at start of follow-up**	Per 10-year increase	1.06 (1.05-1.07)	1.01 (0.99-1.03)	1.07 (1.05-1.08)	0.98 (0.97-1.00)
	**Sex (ref female)**	Male	0.92 (0.89-0.95)	1.16 (1.11-1.22)	1.02 (0.98-1.06)	1.18 (1.14-1.23)
	**Ethnicity (ref white)**	Asian	1.25 (1.13-1.38)	0.88 (0.77-1.00)	1.39 (1.24-1.55)	0.77 (0.68-0.87)
		Black	1.10 (1.02-1.20)	0.91 (0.78-1.06)	1.24 (1.11-1.39)	0.85 (0.77-0.94)
		Mixed	1.00 (0.87-1.15)	0.78 (0.63-0.97)	0.95 (0.83-1.10)	0.90 (0.78-1.03)
		Other	0.78 (0.68-0.89)	1.53 (1.32-1.78)	0.82 (0.71-0.95)	1.38 (1.20-1.58)
		Missing	0.75 (0.71-0.80)	2.36 (2.14-2.60)	0.82 (0.77-0.88)	1.79 (1.68-1.90)
	**Country (ref England)**	Northern Ireland	0.91 (0.69-1.21)	0.49 (0.40-0.61)	1.08 (0.82-1.42)	0.68 (0.51-0.89)
		Scotland	1.10 (0.98-1.24)	0.99 (0.87-1.13)	1.09 (0.95-1.25)	0.99 (0.88-1.11)
		Wales	0.83 (0.74-0.93)	0.69 (0.60-0.79)	1.05 (0.94-1.18)	0.58 (0.51-0.67)
	**SMI diagnosis (ref bipolar disorder)**	Schizophrenia	1.12 (1.08-1.17)	0.97 (0.91-1.03)	1.22 (1.16-1.27)	0.95 (0.91-1.00)
		Other psychoses	0.89 (0.86-0.93)	1.13 (1.06-1.21)	0.99 (0.95-1.04)	1.24 (1.19-1.30)
	**In period variables**	**Exception reported**	0.40 (0.38-0.42)	1.75 (1.64-1.88)	0.56 (0.54-0.59)	1.60 (1.53-1.68)
		**Other QOF register[a]**	1.64 (1.58-1.70)	0.59 (0.55-0.62)	1.99 (1.90-2.08)	0.68 (0.65-0.70)
		**On antipsychotics/mood stabilisers**	1.81 (1.73-1.89)	0.34 (0.32-0.36)	1.59 (1.51-1.67)	0.50 (0.48-0.53)
		**Time since diagnosis**	1.00 (1.00-1.00)	1.00 (1.00-1.00)	1.00 (1.00-1.00)	1.00 (1.00-1.00)
		**Time since registration**	1.00 (1.00-1.00)	1.00 (1.00-1.00)	1.00 (1.00-1.00)	1.00 (1.00-1.00)
		**Years of follow-up**	1.00 (1.00-1.00)	1.00 (1.00-1.00)	1.00 (1.00-1.00)	1.00 (1.00-1.00)
		Year 2	4.81 (0.47-48.80)	5.62 (0.46-68.08)	1.08 (0.38-3.09)	2.66 (1.10-6.46)

Reference: Irregular screening			April 2011 – March 2014 (n=85,274)		April 2014 – March 2018 (n=94,216)	
			Complete (n=12,616, 14.79%)<br>OR (95% CI)	None (n=3,204, 3.76%)<br>OR (95% CI)	Complete (n=12,616, 14.79%)<br>OR (95% CI)	None (n=3,204, 3.76%)<br>OR (95% CI)
	**Year of end of follow-up in period (ref final year)**	Year 3	NA	NA	1.47 (1.36-1.60)	1.83 (1.69-1.99)
**Alcohol**	**Age at start of follow-up**	**Per 10-year increase**	1.07 (1.06-1.08)	1.00 (0.98-1.02)	1.12 (1.09-1.13)	0.97 (0.95-0.99)
	**Sex (ref female)**	Male	1.07 (1.03-1.10)	1.04 (1.00-1.10)	1.01 (0.98-1.04)	1.05 (1.00-1.10)
	**Ethnicity (ref white)**	Asian	1.23 (1.11-1.35)	0.93 (0.81-1.07)	1.33 (1.19-1.48)	0.74 (0.63-0.86)
		Black	1.04 (0.95-1.13)	0.84 (0.72-0.97)	1.11 (1.00-1.22)	0.92 (0.81-1.03)
		Mixed	0.93 (0.80-1.07)	0.91 (0.72-1.13)	1.03 (0.90-1.17)	0.94 (0.76-1.15)
		Other	0.81 (0.71-0.92)	1.57 (1.34-1.84)	0.95 (0.82-1.10)	1.35 (1.13-1.61)
		Missing	0.74 (0.70-0.79)	2.25 (2.04-2.48)	0.80 (0.75-0.85)	1.58 (1.47-1.70)
	**Country (ref England)**	Northern Ireland	0.79 (0.57-1.09)	0.53 (0.41-0.68)	0.27 (0.20-0.35)	0.48 (0.38-0.61)
		Scotland	1.13 (0.99-1.29)	0.94 (0.82-1.08)	0.21 (0.18-0.25)	1.72 (1.48-2.00)
		Wales	0.75 (0.65-0.86)	0.67 (0.59-0.77)	0.19 (0.16-0.23)	0.97 (0.85-1.11)
	**SMI diagnosis (ref bipolar disorder)**	Schizophrenia	1.14 (1.09-1.19)	0.79 (0.74-0.84)	1.13 (1.09-1.19)	0.79 (0.73-0.85)
		Other psychoses	0.95 (0.91-0.99)	1.09 (1.02-1.16)	0.95 (0.91-0.99)	1.15 (1.08-1.23)
	**In period variables**	**Exception reported**	0.40 (0.38-0.42)	1.45 (1.35-1.55)	0.33 (0.31-0.35)	1.08 (1.01-1.15)
		**Other QOF register[a]**	1.46 (1.41-1.52)	0.60 (0.56-0.63)	1.44 (1.39-1.49)	0.65 (0.62-0.69)
		**On antipsychotics/mood stabilisers**	1.72 (1.65-1.80)	0.29 (0.28-0.31)	2.27 (2.16-2.38)	0.37 (0.35-0.39)
		**Time since diagnosis**	1.00 (1.00-1.00)	1.00 (1.00-1.00)	1.00 (1.00-1.00)	1.00 (1.00-1.00)
		**Time since registration**	1.00 (1.00-1.00)	1.00 (1.00-1.00)	1.00 (1.00-1.00)	1.00 (1.00-1.00)

Reference: Irregular screening			April 2011 – March 2014 (n=85,274)		April 2014 – March 2018 (n=94,216)	
			**Complete (n=12,616, 14.79%) OR (95% CI)**	**None (n=3,204, 3.76%) OR (95% CI)**	**Complete (n=12,616, 14.79%) OR (95% CI)**	**None (n=3,204, 3.76%) OR (95% CI)**
		**Years of follow-up**	1.00 (1.00-1.00)	1.00 (1.00-1.00)	1.00 (1.00-1.00)	1.00 (1.00-1.00)
	**Year of end of follow-up in period (ref final year)**	Year 2	0.79 (0.26-2.42)	1.82 (0.28-11.71)	0.59 (0.25-1.37)	1.54 (0.55-4.32)
		Year 3	NA	NA	1.43 (1.31-1.56)	2.47 (2.24-2.73)
**Smoking**	**Age at start of follow-up**	Per 10-year increase	0.96 (0.95-0.97)	1.16 (1.14-1.18)	0.91 (0.90-0.92)	1.15 (1.13-1.17)
	**Sex (ref female)**	Male	1.09 (1.05-1.12)	0.92 (0.88-0.97)	1.25 (1.21-1.29)	0.98 (0.93-1.03)
	**Ethnicity (ref white)**	Asian	0.75 (0.69-0.83)	1.32 (1.15-1.50)	0.66 (0.60-0.72)	1.04 (0.91-1.19)
		Black	0.81 (0.75-0.87)	1.10 (0.95-1.27)	0.79 (0.72-0.87)	1.18 (1.05-1.33)
		Mixed	0.87 (0.77-0.99)	0.84 (0.64-1.09)	0.95 (0.85-1.06)	0.97 (0.78-1.21)
		Other	0.77 (0.68-0.89)	1.67 (1.39-2.00)	0.87 (0.78-0.98)	1.31 (1.09-1.58)
		Missing	0.76 (0.71-0.80)	2.18 (1.95-2.45)	0.82 (0.78-0.87)	1.55 (1.44-1.67)
	**Country (ref England)**	Northern Ireland	0.98 (0.75-1.26)	0.46 (0.36-0.58)	0.32 (0.24-0.44)	0.62 (0.49-0.79)
		Scotland	1.19 (1.07-1.33)	0.67 (0.58-0.78)	0.35 (0.30-0.40)	1.11 (0.98-1.25)
		Wales	0.96 (0.86-1.08)	0.58 (0.49-0.68)	0.36 (0.32-0.41)	0.72 (0.63-0.84)
	**SMI diagnosis (ref bipolar disorder)**	Schizophrenia	1.20 (1.16-1.25)	0.94 (0.88-1.00)	1.27 (1.22-1.32)	0.88 (0.83-0.95)
		Other psychoses	0.95 (0.92-0.99)	1.00 (0.93-1.07)	0.99 (0.95-1.02)	1.11 (1.05-1.18)
	**In period variables**	**Exception reported**	0.64 (0.62-0.67)	1.28 (1.19-1.38)	0.75 (0.72-0.78)	1.21 (1.14-1.28)
		**Other QOF register[a]**	1.68 (1.62-1.74)	0.61 (0.57-0.65)	1.52 (1.46-1.57)	0.64 (0.61-0.68)
		**On antipsychotics/mood stabilisers**	1.52 (1.46-1.59)	0.44 (0.41-0.48)	1.58 (1.52-1.65)	0.60 (0.56-0.63)
		**Time since diagnosis**	1.00 (1.00-1.00)	1.00 (1.00-1.00)	1.00 (1.00-1.00)	1.00 (1.00-1.00)
		**Time since registration**	1.00 (1.00-1.00)	1.00 (1.00-1.00)	1.00 (1.00-1.00)	1.00 (1.00-1.00)
		**Years of follow-up**	1.00 (1.00-1.00)	1.00 (1.00-1.00)	1.00 (1.00-1.00)	1.00 (1.00-1.00)

Reference: Irregular screening		April 2011 – March 2014 (n=85,274)		April 2014 – March 2018 (n=94,216)	
		**Complete (n=12,616, 14.79%) OR (95% CI)**	**None (n=3,204, 3.76%) OR (95% CI)**	**Complete (n=12,616, 14.79%) OR (95% CI)**	**None (n=3,204, 3.76%) OR (95% CI)**
**Year of end of follow-up in period (ref final year)**	Year 2	0.38 (0.12-1.22)	3.90 (0.83-18.25)	0.64 (0.28-1.45)	2.10 (0.73-6.07)
	Year 3	NA	NA	1.56 (1.46-1.67)	2.43 (2.19-2.69)

OR: Odds ratio; 95% CI: 95% confidence interval; ref: Reference category; SMI: Severe Mental Illness; QOF: Quality and Outcomes Framework; BMI: body mass index
a: Defined as presence on QOF register for atrial fibrillation, coronary heart disease, hypertension, peripheral artery disease, stroke or diabetes.

Table S8: Multinomial logistic regression for the odds ratios of always receiving complete screening or receiving no screening compared to irregular screening of cardiovascular risk factors in patients with severe mental illness with available area-based deprivation (IMD) data (England only), n=40,264

		2011-2014 (n=29,548)		2014-2018 (n=28,762)	
**Reference: Irregular screening**		**Complete (n=4,346, 14.71%)**	**None (n=1,078, 3.65%)**	**Complete (n=2,437, 8.47%)**	**None (n=583, 2.03%)**
		**OR (95% CI)**	**OR (95% CI)**	**OR (95% CI)**	**OR (95% CI)**
**Age at start of follow-up**	Per 10-year increase	1.21 (1.17-1.23)	0.97 (0.90-1.03)	1.16 (1.13-1.20)	0.90 (0.84-0.98)
**Sex (ref female)**	Male	1.22 (1.14-1.31)	1.28 (1.11-1.47)	1.32 (1.21-1.44)	1.48 (1.22-1.81)
**Ethnicity (ref White)**	Asian	1.26 (1.05-1.51)	1.05 (0.72-1.54)	1.56 (1.23-1.98)	0.73 (0.47-1.15)
	Black	0.84 (0.68-1.03)	1.16 (0.81-1.67)	1.20 (0.95-1.53)	0.99 (0.67-1.47)
	Mixed	0.86 (0.63-1.18)	0.83 (0.42-1.62)	0.91 (0.61-1.37)	0.71 (0.33-1.53)
	Other	0.83 (0.67-1.04)	2.46 (1.69-3.60)	0.93 (0.66-1.30)	1.13 (0.66-1.96)
	Missing	0.67 (0.57-0.80)	4.45 (3.21-6.16)	0.73 (0.61-0.86)	2.24 (1.77-2.84)
**SMI diagnosis (ref bipolar disorder)**	Schizophrenia	1.26 (1.15-1.37)	0.91 (0.75-1.09)	1.24 (1.10-1.39)	0.88 (0.69-1.13)
	Other psychoses	0.91 (0.82-1.00)	1.08 (0.90-1.29)	0.97 (0.86-1.09)	1.19 (0.95-1.50)
**In period variables**	**Exception reported**	0.36 (0.31-0.41)	1.24 (0.93-1.64)	0.47 (0.41-0.53)	1.56 (1.26-1.93)
	**Other QOF[a]**	1.95 (1.77-2.16)	0.34 (0.29-0.40)	2.96 (2.54-3.45)	0.32 (0.26-0.39)
	**On antipsychotics/mood stabilisers**	1.84 (1.64-2.06)	0.16 (0.14-0.20)	2.09 (1.82-2.41)	0.22 (0.17-0.27)
	**Time since diagnosis**	1.00 (1.00-1.00)	1.00 (1.00-1.00)	1.00 (1.00-1.00)	1.00 (1.00-1.00)
	**Time since registration**	1.00 (1.00-1.00)	1.00 (1.00-1.00)	1.00 (1.00-1.00)	1.00 (1.00-1.00)
	**Years of follow-up**	1.00 (1.00-1.00)	1.00 (1.00-1.00)	1.00 (1.00-1.00)	1.00 (1.00-1.00)
**Year of end of follow-up in period (ref final year)**	Year 2	0.00 (00.00-0.00)	0.00 (00.00-0.00)	0.00 (00.00-0.00)	8.06 (0.43-150.80)
	Year 3	NA	NA	1.40 (1.16-1.68)	2.81 (2.12-3.73)
**Index of multiple deprivation quintile (ref 1 - least deprived)**	2	1.01 (0.85-1.21)	1.00 (0.77-1.31)	1.08 (0.86-1.35)	0.94 (0.70-1.26)
	3	1.05 (0.88-1.26)	1.03 (0.75-1.40)	1.21 (0.94-1.54)	0.80 (0.60-1.06)
	4	1.25 (1.05-1.50)	0.96 (0.61-1.52)	1.37 (1.07-1.77)	0.75 (0.56-1.01)
	5 – Most deprived	1.27 (1.05-1.54)	1.16 (0.81-1.65)	1.61 (1.23-2.09)	0.74 (0.56-0.99)

OR: Odds ratio; 95% CI: 95% confidence interval; ref: Reference category; SMI: Severe Mental Illness; QOF: Quality and Outcomes Framework. a: Defined as presence on QOF register for atrial fibrillation, coronary heart disease, hypertension, peripheral artery disease, stroke or diabetes.

Table S9: Multinomial logistic regression for the odds ratios for always receiving complete screening or receiving no screening compared to irregular screening for cardiovascular risk factors in patients active for the full duration of each time period, n=83,838

		2011-2014 (66,816)		2014-2018 (61,003)	
**Reference: Irregular screening**		**Complete (n=9743, 14.58%)**	**None (n=1742, 2.61%))**	**Complete (n=4708, 7.72%)**	**None (n=1120, 1.84%)**
		**OR (95% CI)**	**OR (95% CI)**	**OR (95% CI)**	**OR (95% CI)**
**Age at start of follow-up**	Per 10-year increase	1.20 (1.17-1.21)	0.94 (0.90-0.99)	1.20 (1.17-1.23)	0.87 (0.82-0.92)
**Sex (ref female)**	Male	1.24 (1.18-1.30)	1.30 (1.17-1.45)	1.37 (1.28-1.46)	1.47 (1.28-1.68)
**Ethnicity (ref white)**	Asian	1.27 (1.09-1.46)	0.92 (0.66-1.28)	1.85 (1.54-2.23)	0.89 (0.59-1.35)
	Black	0.89 (0.77-1.03)	1.30 (0.96-1.77)	1.34 (1.10-1.63)	1.46 (1.08-1.96)
	Mixed	0.76 (0.61-0.95)	1.10 (0.68-1.77)	1.16 (0.87-1.54)	0.75 (0.40-1.42)
	Other	0.78 (0.64-0.96)	2.43 (1.80-3.27)	0.89 (0.65-1.20)	1.76 (1.17-2.63)
	Missing	0.68 (0.62-0.76)	3.69 (3.19-4.28)	0.71 (0.62-0.82)	2.51 (2.12-2.97)
**Country (ref England)**	NI	1.16 (0.76-1.76)	0.48 (0.33-0.70)	0.66 (0.40-1.08)	0.79 (0.47-1.31)
	Scotland	1.61 (1.34-1.94)	0.96 (0.77-1.21)	1.23 (0.95-1.59)	2.39 (1.92-2.97)
	Wales	0.85 (0.70-1.04)	0.79 (0.59-1.05)	0.54 (0.42-0.69)	1.38 (0.98-1.94)
**SMI diagnosis (ref bipolar disorder)**	Schizophrenia	1.29 (1.21-1.36)	0.98 (0.85-1.13)	1.38 (1.28-1.50)	1.04 (0.86-1.25)
	Other psychoses	0.95 (0.90-1.01)	1.21 (1.06-1.38)	1.02 (0.94-1.11)	1.36 (1.15-1.61)
**In period variables**	**Exception reported**	0.39 (0.36-0.42)	1.61 (1.41-1.85)	0.50 (0.46-0.54)	1.61 (1.38-1.87)
	**Other QOF register**[a]	2.03 (1.90-2.18)	0.30 (0.27-0.34)	3.97 (3.45-4.57)	0.27 (0.23-0.31)
	**On antipsychotics/mood stabilisers**	1.98 (1.83-2.13)	0.18 (0.16-0.20)	2.01 (1.81-2.24)	0.19 (0.16-0.22)
	**Time since diagnosis**	1.00 (1.00-1.00)	1.00 (1.00-1.00)	1.00 (1.00-1.00)	1.00 (1.00-1.00)
	**Time since registration**	1.00 (1.00-1.00)	1.00 (1.00-1.00)	1.00 (1.00-1.00)	1.00 (1.00-1.00)

OR: Odds ratio; 95% CI: 95% confidence interval; ref: Reference category; SMI: Severe Mental Illness; QOF: Quality and Outcomes Framework.
a: Defined as presence on QOF register for atrial fibrillation, coronary heart disease, hypertension, peripheral artery disease, stroke or diabetes.

Figure S1: Study population flow diagram, detailing inclusion and exclusion criteria

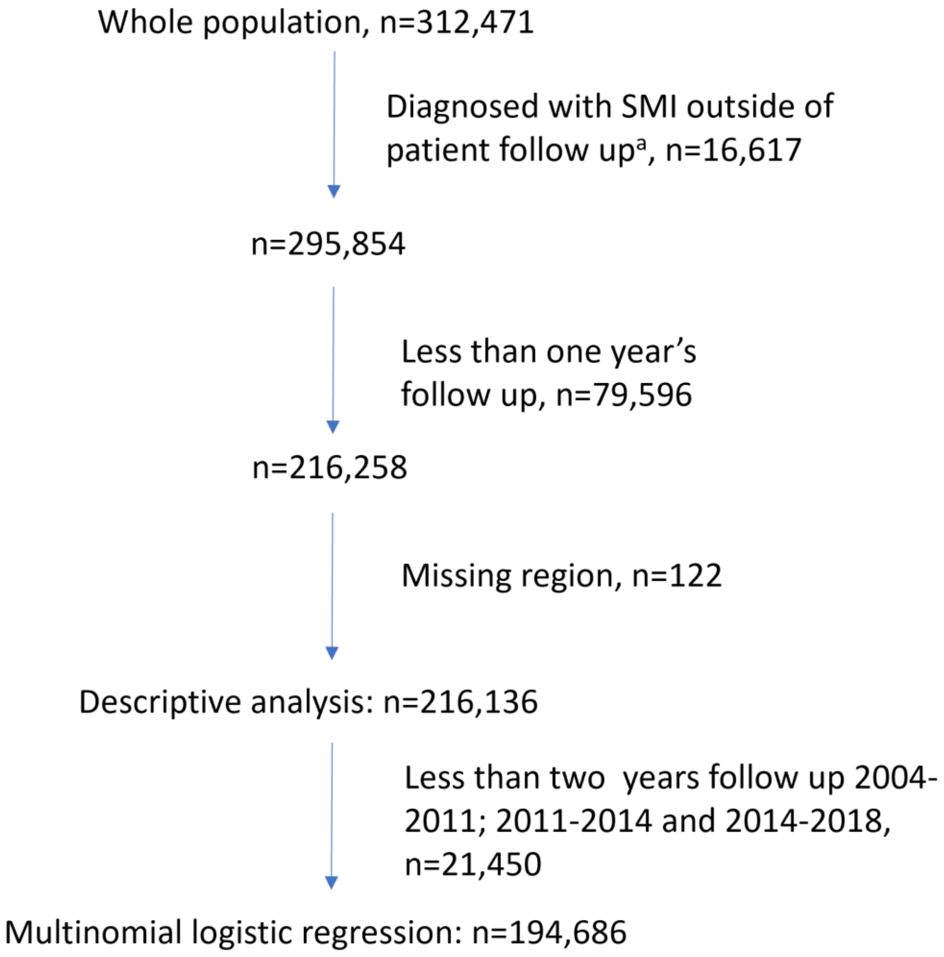

a: After follow-up ends (earliest of death, leaving the primary care practice, age 100 or last data collection by CPRD) or under 18.

Figure S2: Histogram of follow up time for the study cohort

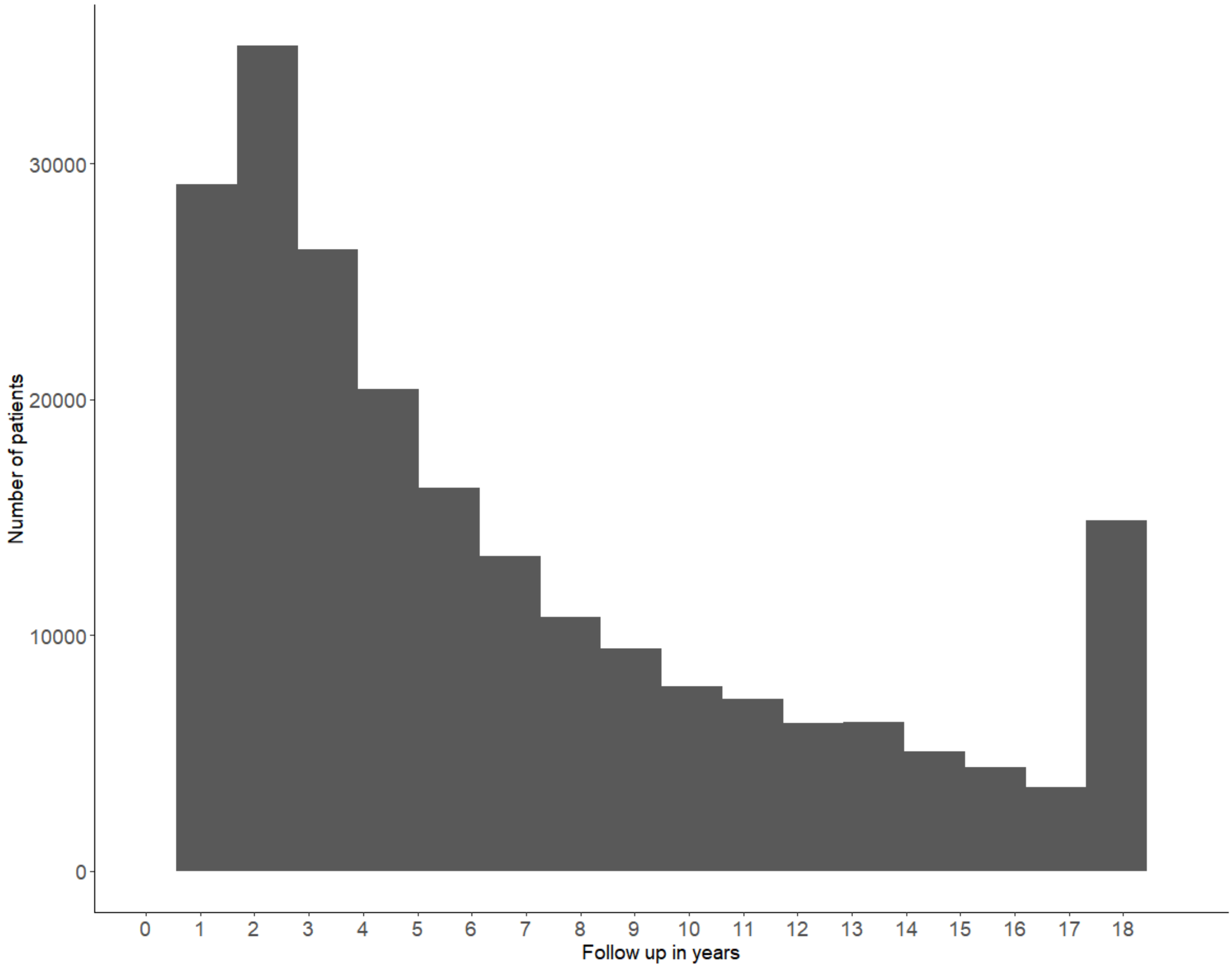

Figure S3: Cardiovascular risk factor screening prevalence in patients with severe mental illness, by age

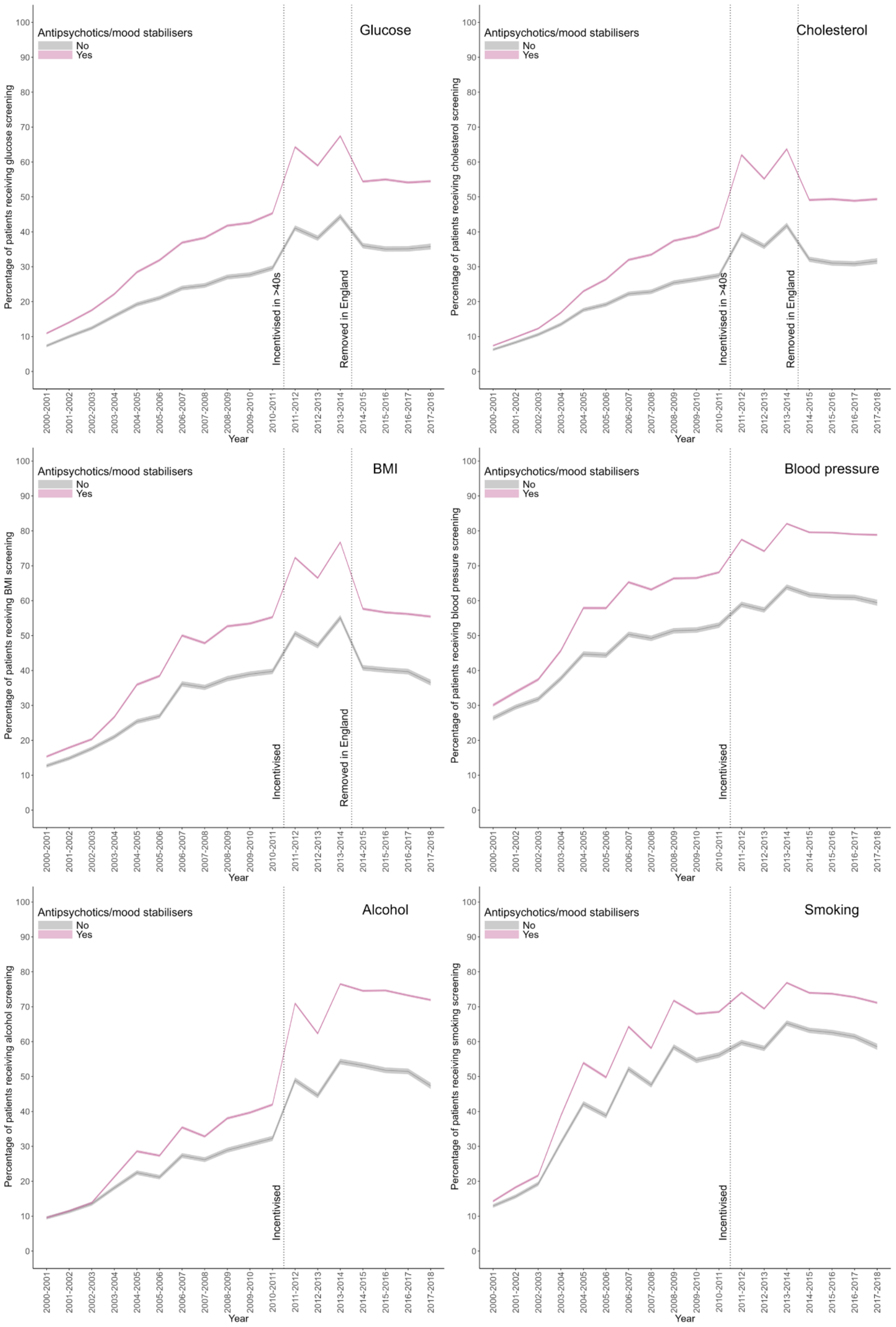

Figure S4: Cardiovascular risk factor screening prevalence in people with severe mental illness, by country*

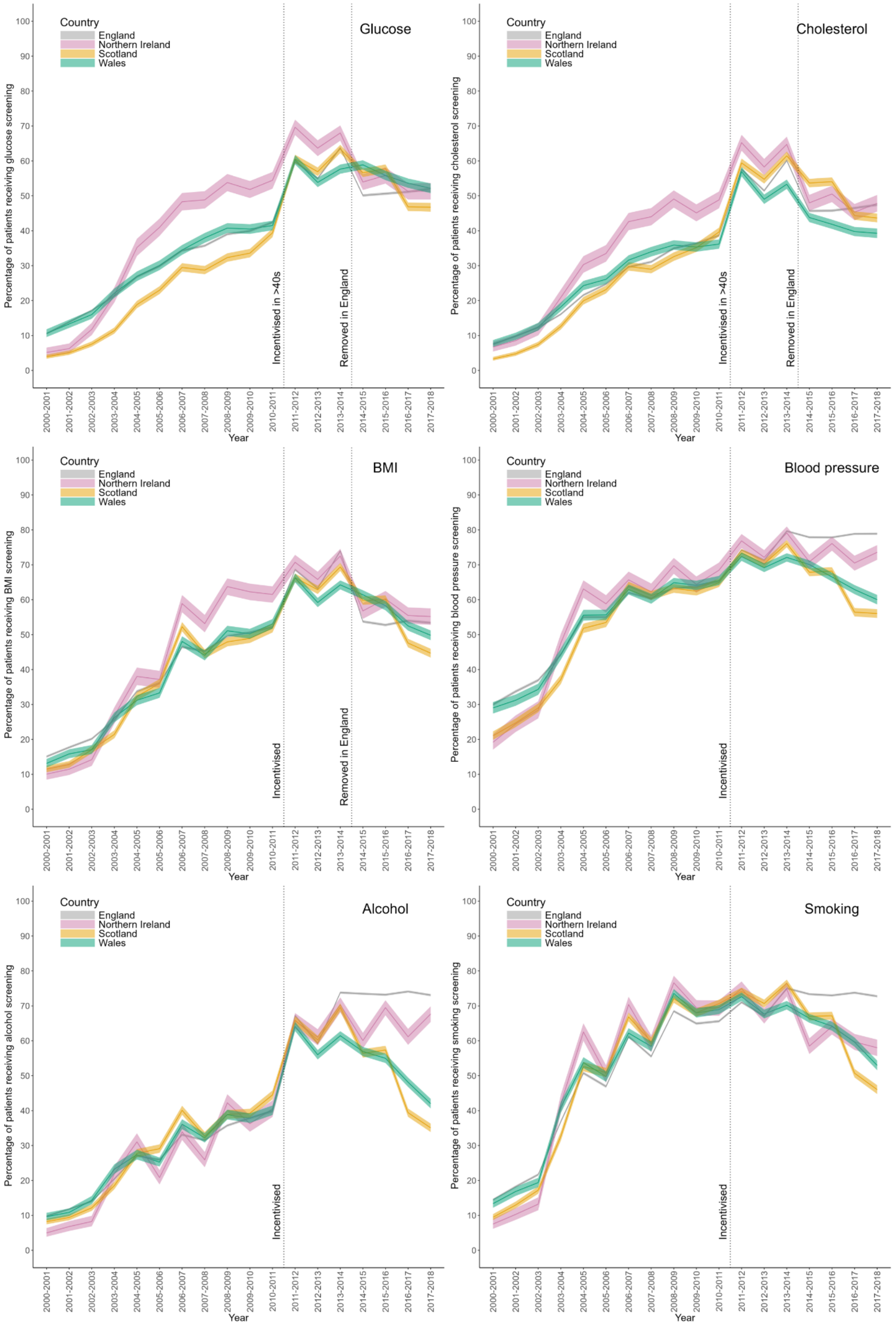

*From 2011-2012 incentivisation differs by country. See Table S1 for further explanation

Figure S5: Cardiovascular risk factor screening prevalence in people with severe mental illness, by current severe mental illness diagnosis

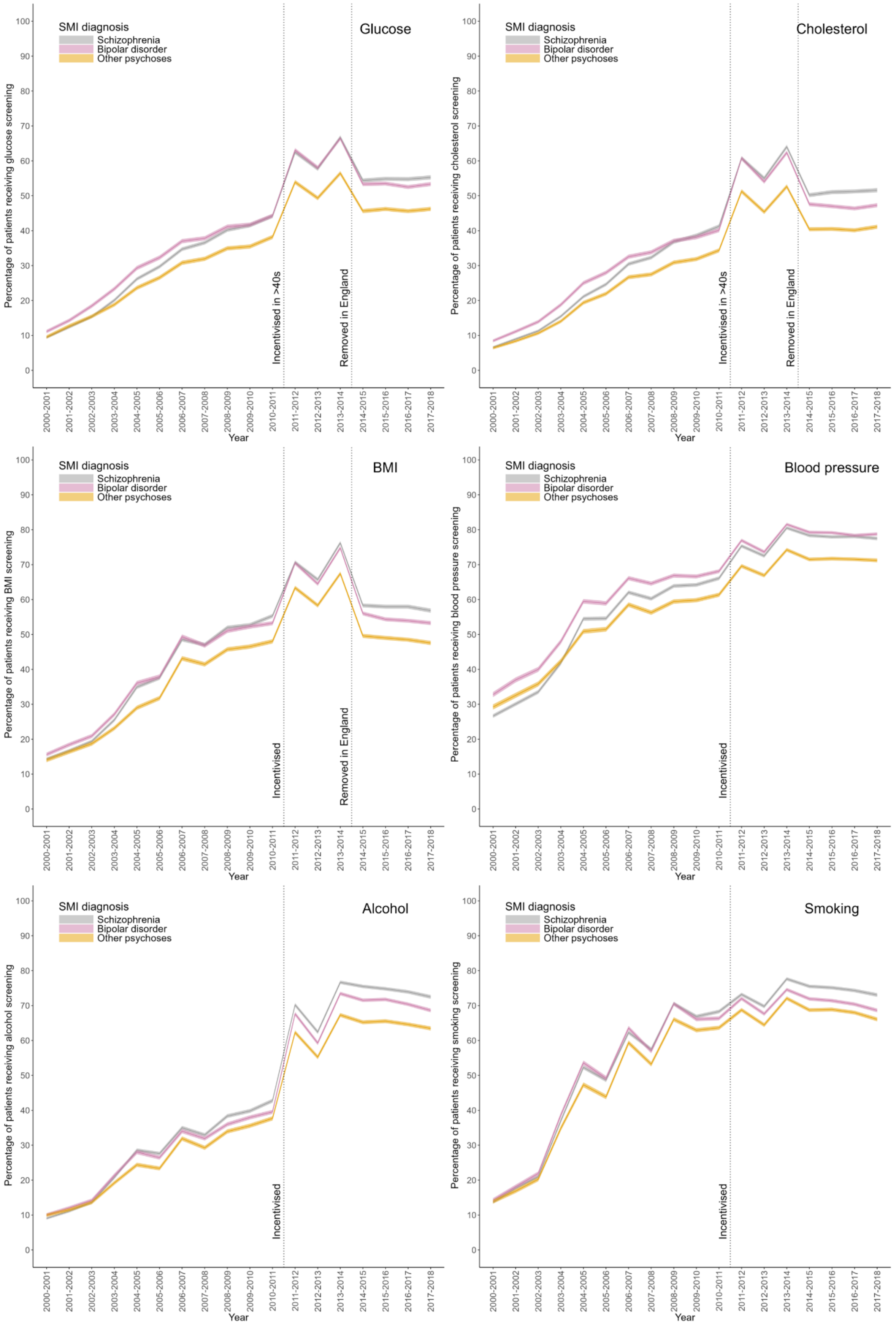

Figure S6: Cardiovascular risk factor screening prevalence in people with severe mental illness, by sex

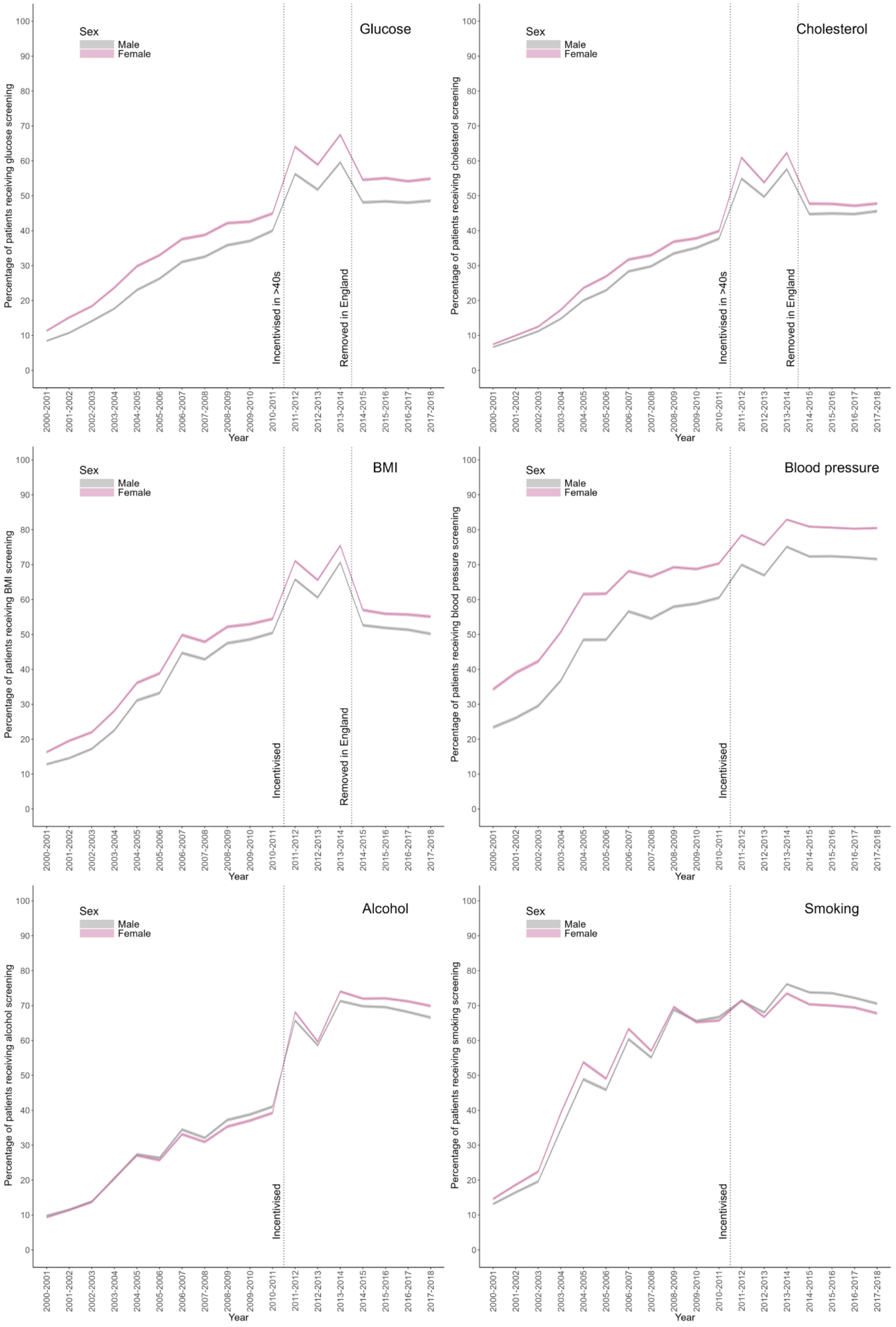

Figure S7: Cardiovascular risk factor screening prevalence in people with severe mental illness, by presence on another QOF register that incentivises cardiovascular risk factor screening

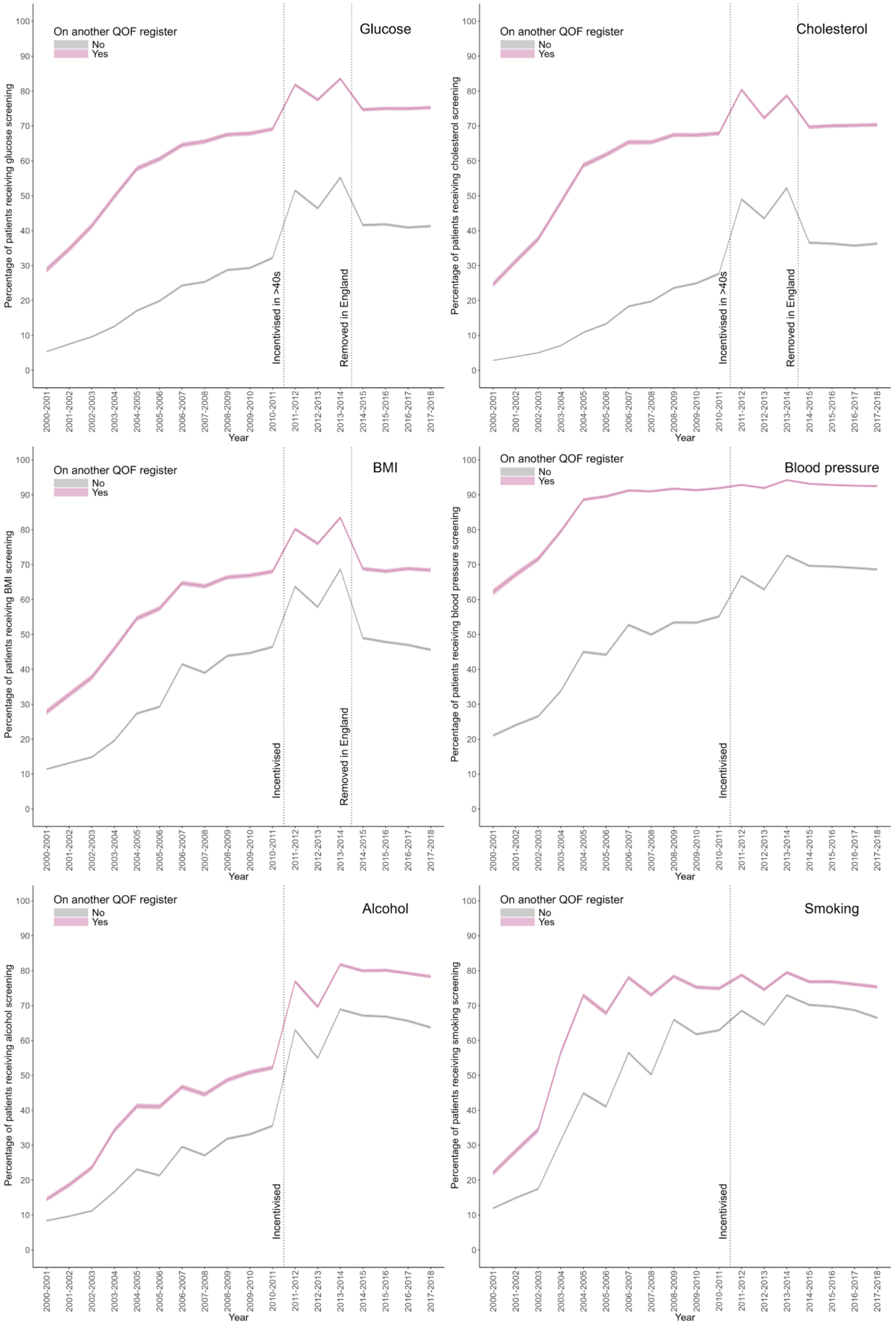

Figure S8: Cardiovascular risk factor screening prevalence in people with severe mental illness, by prescription of antipsychotics or mood stabilisers

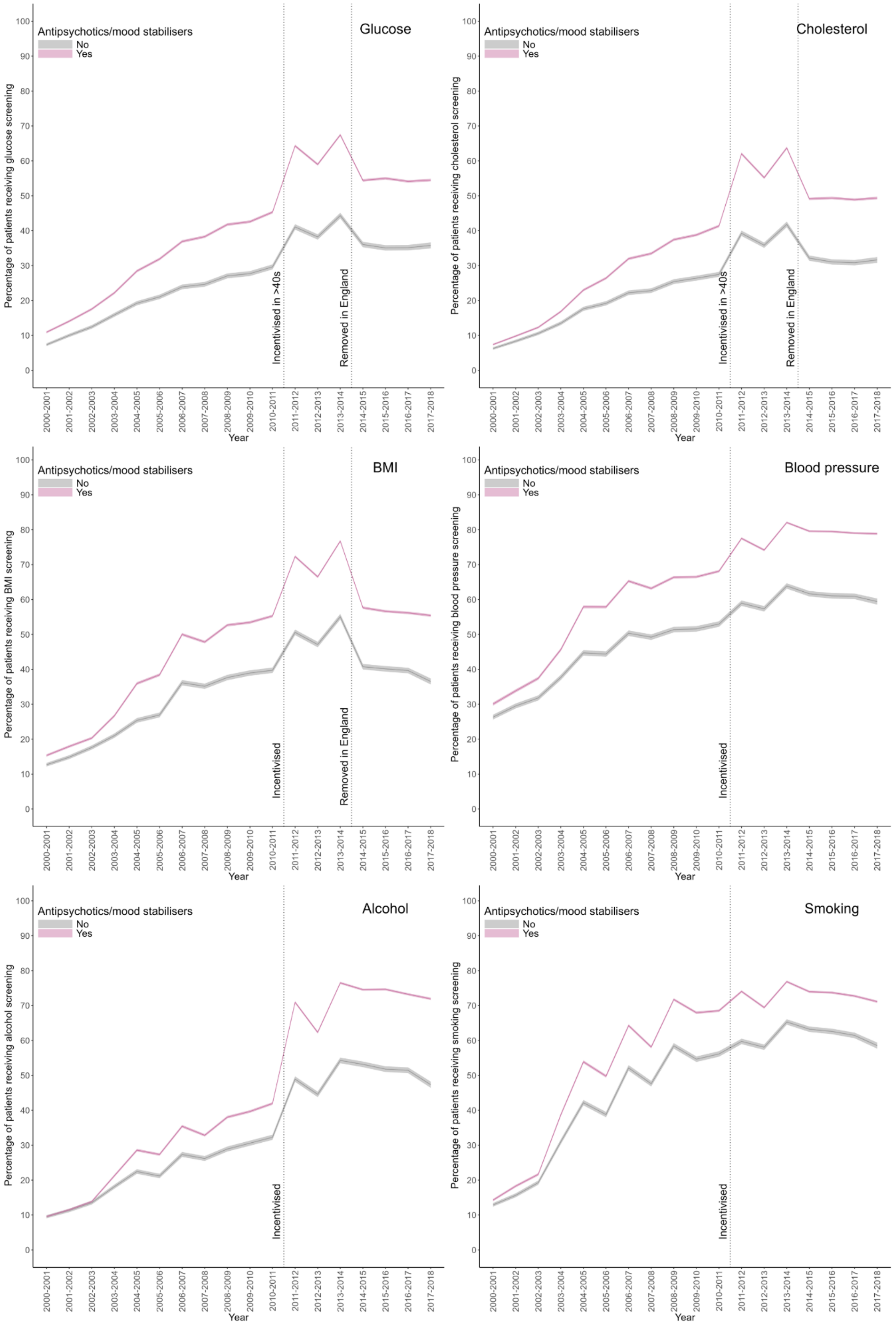

Figure S9: Cardiovascular risk factor screening in patients with severe mental illness, by ethnicity

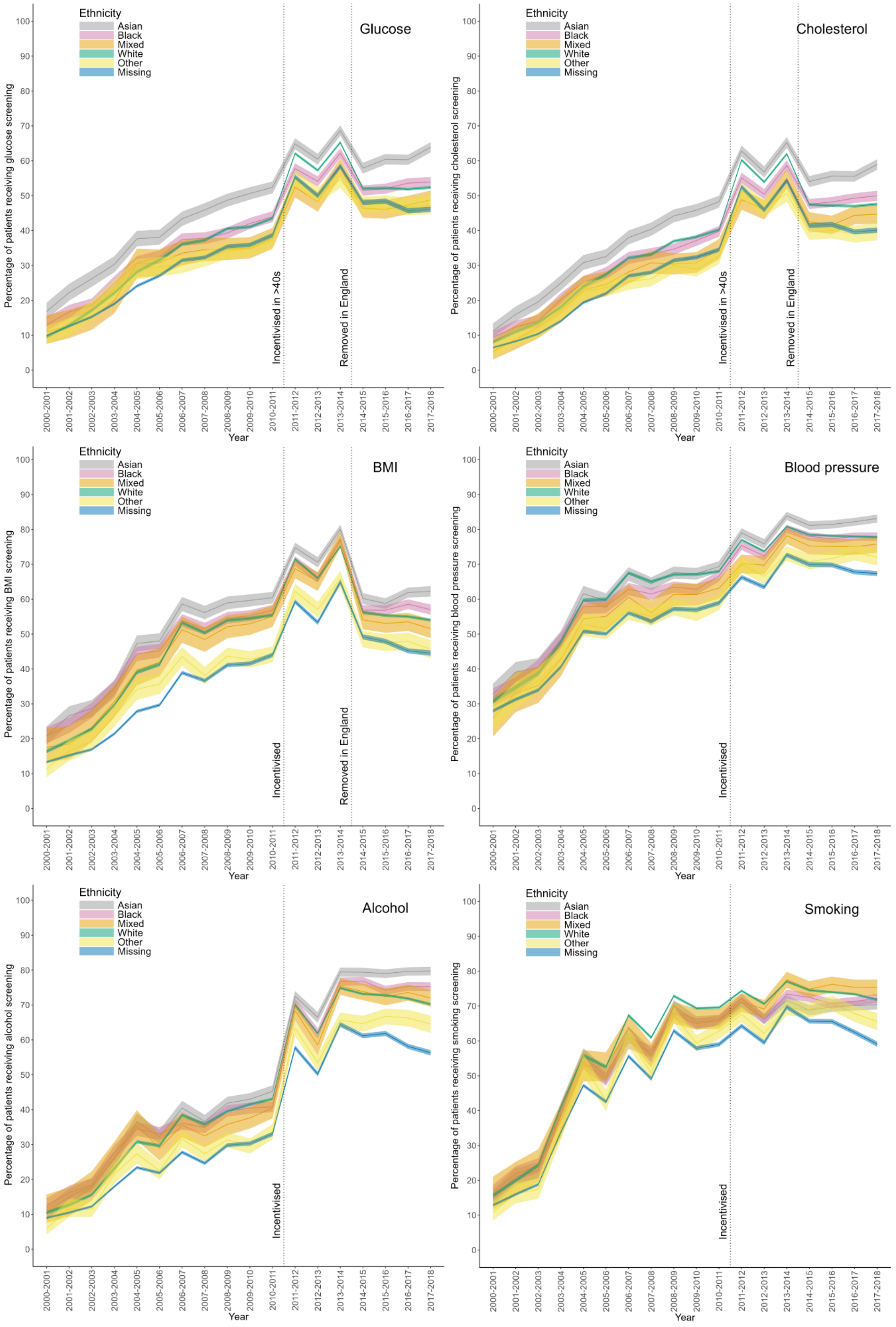

Figure S10: Cardiovascular risk factor screening prevalence in patients with severe mental illness, by exception reporting status that year*

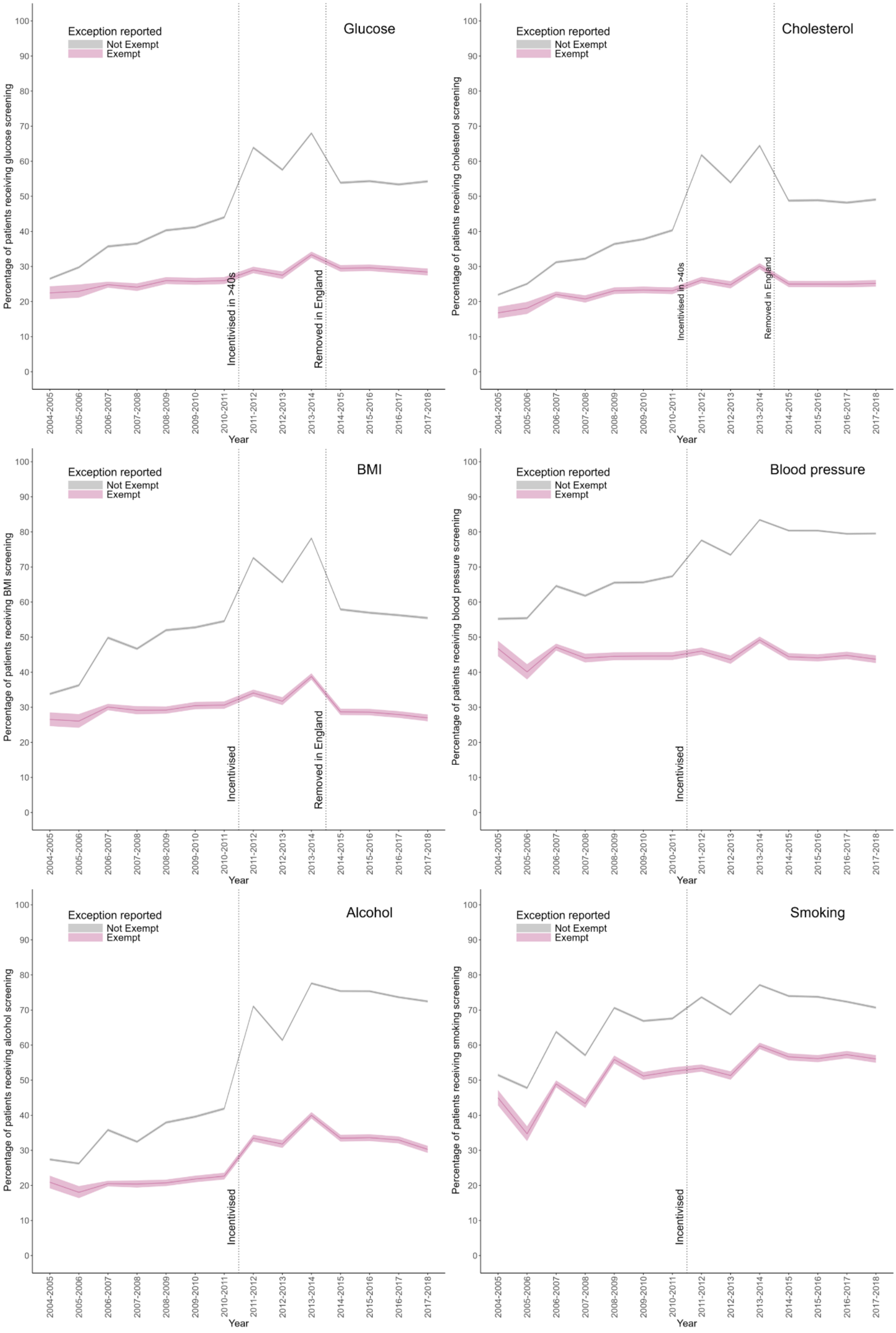

*Limited to 2004 onwards when exception reporting was introduced

Figure S11: Cardiovascular risk factor screening prevalence in people with severe mental illness, by index of multiple deprivation for patients in England where this is available (n=137,941)*

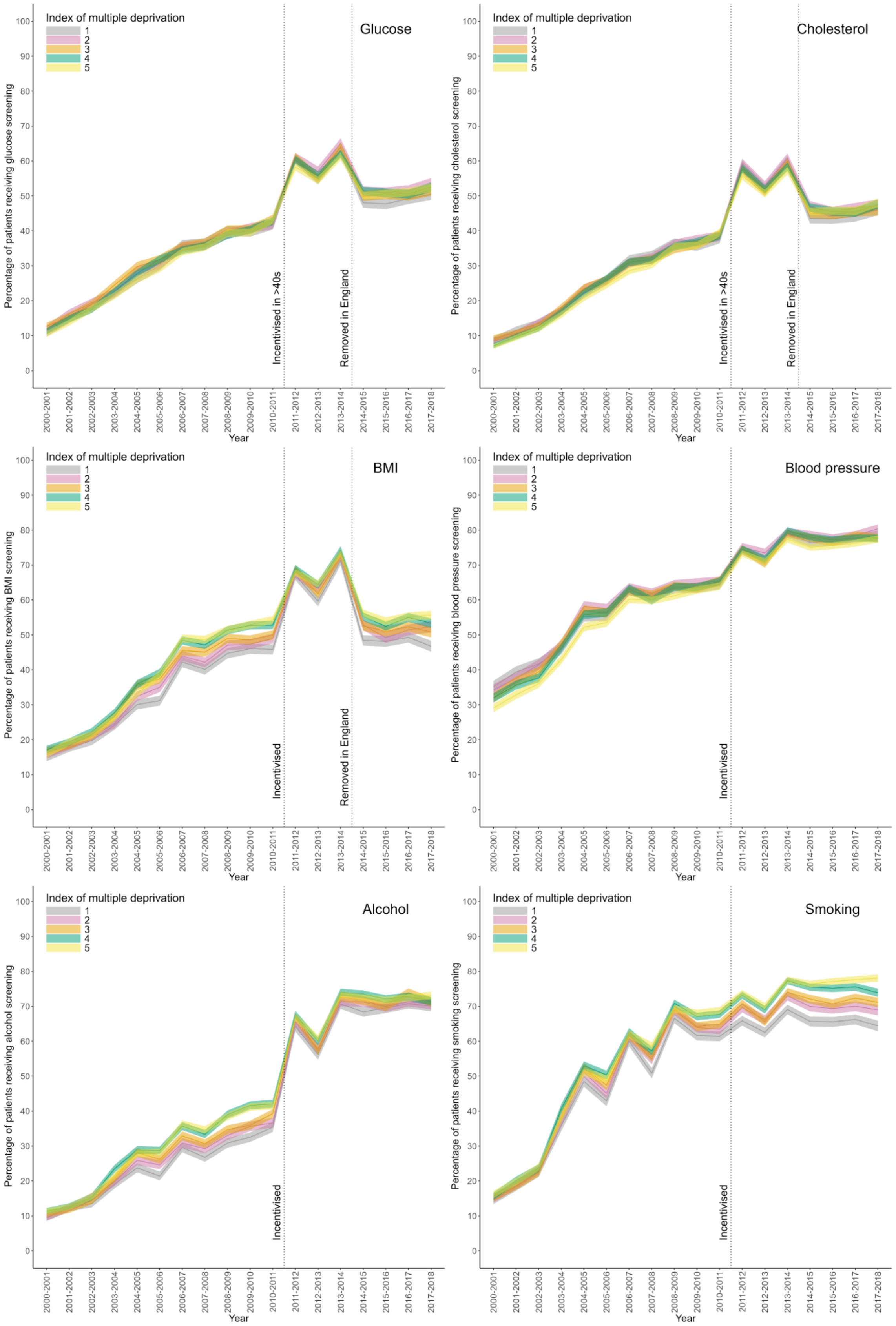

*

1=least deprived; 5=most deprived.
